# Time-Resolved Imaging of Single HIV-1 Uncoating *In Vitro* and in Living Cells

**DOI:** 10.1371/journal.ppat.1005709

**Published:** 2016-06-20

**Authors:** Ashwanth C. Francis, Mariana Marin, Jiong Shi, Christopher Aiken, Gregory B. Melikyan

**Affiliations:** 1 Department of Pediatric, Emory University School of Medicine, Atlanta, Georgia, United States of America; 2 Department of Pathology, Microbiology and Immunology, Vanderbilt University Medical Center. Nashville, Tennessee, United States of America; 3 Children’s Healthcare of Atlanta, Atlanta, Georgia, United States of America; Fred Hutchinson Cancer Research Center, UNITED STATES

## Abstract

Disassembly of the cone-shaped HIV-1 capsid in target cells is a prerequisite for establishing a life-long infection. This step in HIV-1 entry, referred to as uncoating, is critical yet poorly understood. Here we report a novel strategy to visualize HIV-1 uncoating using a fluorescently tagged oligomeric form of a capsid-binding host protein cyclophilin A (CypA-DsRed), which is specifically packaged into virions through the high-avidity binding to capsid (CA). Single virus imaging reveals that CypA-DsRed remains associated with cores after permeabilization/removal of the viral membrane and that CypA-DsRed and CA are lost concomitantly from the cores *in vitro* and in living cells. The rate of loss is modulated by the core stability and is accelerated upon the initiation of reverse transcription. We show that the majority of single cores lose CypA-DsRed shortly after viral fusion, while a small fraction remains intact for several hours. Single particle tracking at late times post-infection reveals a gradual loss of CypA-DsRed which is dependent on reverse transcription. Uncoating occurs both in the cytoplasm and at the nuclear membrane. Our novel imaging assay thus enables time-resolved visualization of single HIV-1 uncoating in living cells, and reveals the previously unappreciated spatio-temporal features of this incompletely understood process.

## Author Summary

HIV-1 genome and key enzymes required for establishing productive infection are encased in a cone-shaped shell made of the capsid protein (CA). After being released into the cytosol of target cells, the cone-shaped core complex undergoes a series of carefully orchestrated steps, including uncoating (loss of CA). HIV-1 uncoating remains poorly understood, due in part to the lack of direct assays enabling studies of this process in living cells. Here, we introduce a novel strategy for labeling the HIV-1 capsid without genetically modifying the CA protein. We designed a novel fluorescent cyclophilin A construct that binds the capsid with an extremely high avidity and (1) efficiently incorporates into virions without compromising infectivity; (2) remains bound to cores after viral fusion; and (3) is lost from post-fusion cores along with CA. The novel imaging assay provides new insights into the kinetics and spatial distribution of HIV-1 uncoating in living cells.

## Introduction

Mature HIV-1 particles contain a cone-shape capsid core made of a hexagonal lattice of the capsid protein (CA) that encases the viral genomic RNA, nucleocapsid (NC), reverse transcriptase (RT) and integrase (IN) proteins. After HIV-1 fuses with a target cell, the released viral cores go through a series of carefully orchestrated steps that ultimately lead to productive infection. A key early step of HIV-1 entry—referred to as uncoating—is generally defined as (full or partial) shedding of CA from the viral core (reviewed in [1–3]). Several lines of genetic and functional evidence support the importance of this incompletely understood process in regulating reverse transcription and nuclear import of pre-integration complexes (PICs) (reviewed in [1–3]). Given its critical role in productive entry, the HIV-1 capsid is considered an attractive target for the development of new antiviral drugs [1,4].

HIV-1 uncoating has been traditionally studied by biochemical assays [5–8], which revealed a general correlation between capsid stability and infectivity, supporting the importance of timely uncoating [5]. Establishment of the fate-of-capsid assay [6,7,9], which determines the fraction of particulate capsid recovered from cells shortly after infection, allowed assessment of the effects of host factors and pharmacological agents on the core stability. However, most of these assays examine the bulk population of viruses, many of which are not infectious (reviewed in [1]). The more recently developed cyclosporine A (CsA) washout assay [8] enables indirect measurements of HIV-1 uncoating based upon the virus escape from the host restriction factor, TRIMCyp. However, this assay does not provide information regarding the sites of HIV-1 uncoating, and the interpretation of the obtained results is complex [1].

To elucidate the sites of HIV-1 uncoating, a complementary *in-situ* uncoating assay has been introduced [8]. This assay visualizes the loss of immunolabeled CA/p24 from the eGFP-Vpr labeled HIV-1 reverse transcription complexes (RTC)/PICs in fixed cells after infection. The inability to follow the dynamics of HIV-1 uncoating in fixed cells underscores the need for live cell imaging techniques.

Direct visualization of capsid uncoating in living cells requires the labeling of both RTC/PIC and CA. Whereas PICs have been visualized by incorporating an integrase-GFP (IN-GFP) chimera into pseudoparticles [10,11], attempts to label CA were not successful. CA labeling with a fluorescent protein or a smaller tetracysteine tag [12] adversely affects virus infectivity. Even point mutations in CA can compromise infectivity through altering capsid assembly and/or stability (e.g., [5]). A recently introduced microscopy assay that may indirectly monitor HIV-1 uncoating live cells is based upon the assertion that a fraction of GFP molecules produced upon cleavage of Gag-iGFP precursor [13] is trapped within an intact mature core and is released at the time of uncoating [14,15]. However, further validation of the notion that a detectable number of GFP molecules is trapped in a considerable fraction of mature cores is needed.

Here, we developed a novel assay to visualize single HIV-1 uncoating in living cells using the oligomeric cyclophilin A-DsRed chimera (CypA-DsRed) as a novel CA probe. Single virus imaging reveals that this probe efficiently and specifically incorporates into HIV-1 particles without noticeably compromising viral infectivity. Unlike the monomeric version of CypA tagged with a fluorescent protein, CypA-DsRed tightly binds to CA and reports the loss of CA during HIV-1 uncoating. The loss of CypA-DsRed from single cores is delayed upon inhibition of reverse transcription and correlates well with the core stability *in vitro* and in living cells. Importantly, kinetic measurements revealed that the majority of cores uncoat shortly after viral fusion, whereas a small fraction of incoming cores retain CypA-DsRed for several hours. Collectively, our results show that CypA-DsRed enables time-resolved visualization of single HIV-1 uncoating, thus providing an important new tool for understanding the early steps in HIV-1 infection.

## Results

### Oligomeric CypA-DsRed efficiently incorporates into HIV-1 and can functionally compensate for the lack of CypA expression in target cells

In light of the extreme sensitivity of HIV-1 to substitutions and insertions in the CA protein [12,16], we sought to use the CA-binding protein, CypA, as a less invasive means to label the capsid core. CypA specifically incorporates into HIV-1 particles *via* association with the CA region of the Gag polyprotein [17–19]. To assess the efficiency of CypA incorporation into virions, pseudoviruses lacking or containing CypA tagged with different red fluorescent proteins were produced. Fluorescently tagged IN incorporated into virions by co-expressing the Vpr-integrase-superfolderGFP (Vpr-IN-sfGFP [11]) construct was used as a marker for the viral RTC/PICs. CypA/IN colocalization was assessed by imaging single viruses adhered to coverslips. In agreement with the previously published results [20], CypA tagged with mCherry or with DsRed-derived monomeric mRFP [21] is not efficiently incorporated into viral particles (S1A and S1B Fig) and is thus not compatible with single virus imaging. Such inefficient incorporation of labeled CypA, in spite of abundant expression in virus-producing cells (S1C Fig), is consistent with its relatively low affinity (7–16 μM) for CA [22–24].

We reasoned that the CypA recruitment into HIV-1 virions could be improved by increasing its binding avidity. Indeed, a previous study has demonstrated that multimerization of CypA promotes its binding to the HIV-1 capsid [25]. To create oligomeric CypA and make it visible at the same time, we fused CypA in frame with the tetrameric DsRed fluorescent protein from *Discosoma* [26]. Indeed, DsRed and CypA-DsRed, but not mRFP or CypA-mRFP, are preferentially present in an oligomeric form in cell lysates, and these oligomers are converted into monomers after boiling (S2A Fig). This result implies that DsRed drives the oligomerization of CypA, as evidenced by the ~200 kDa band expected for a CypA-DsRed tetramer.

**Fig 1:**
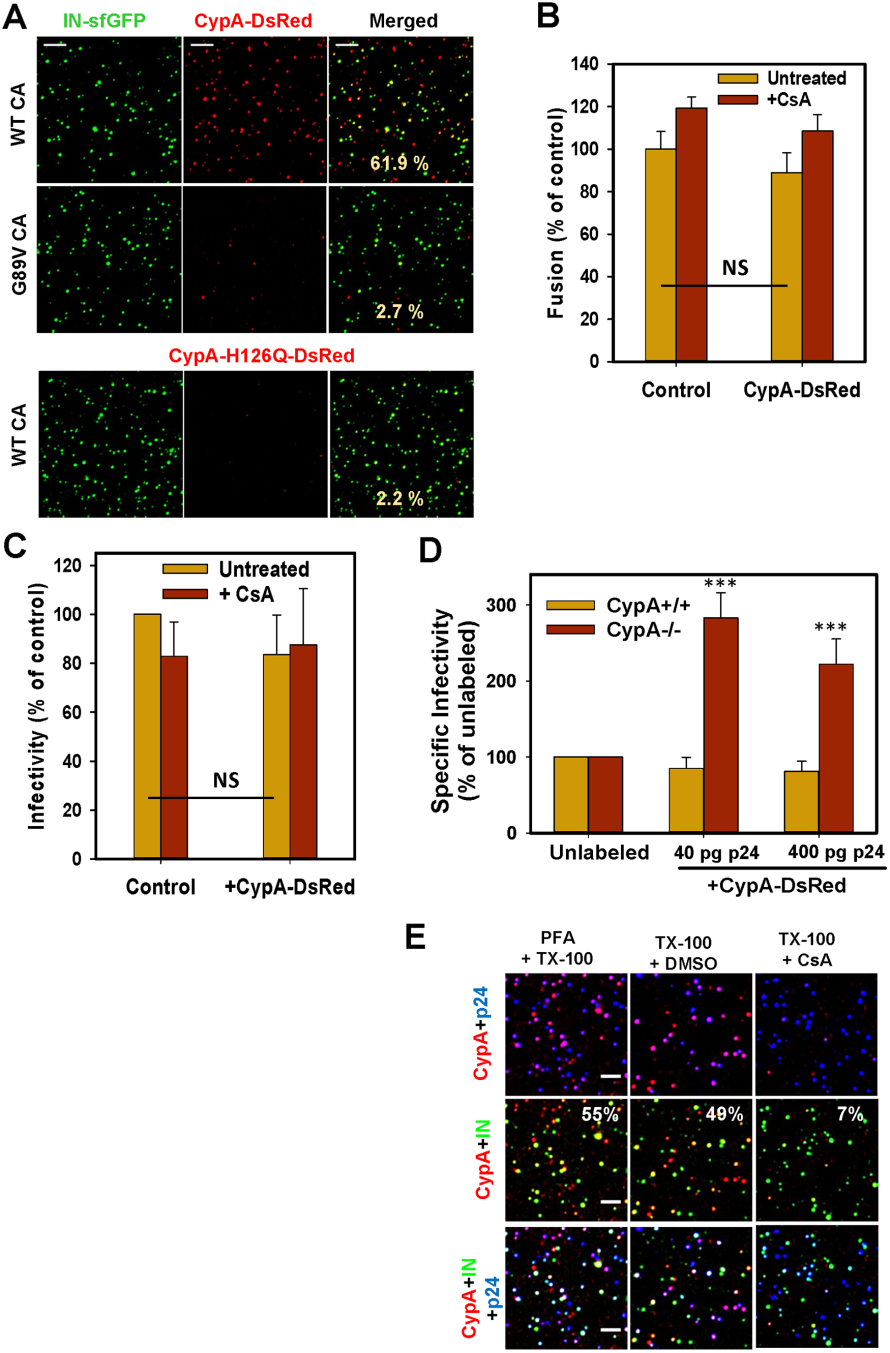
CypA-DsRed specifically incorporates into HIV-1 cores through tight binding to capsid and obviates the need for CypA in target Jurkat cells. (A) VSV-G pseudotyped particles containing WT or G89V CA were co-labeled with IN-sfGFP and CypA-DsRed. Bottom panel: viruses bearing WT-CA and the CypA-DsRed H126Q mutant. (B) TZM-bl cells were inoculated with equal p24 amounts of VSV-G pseudotyped particles containing BlaM-Vpr and containing or lacking CypA-DsRed. Virus fusion activity was measured in the presence or absence of 5 μM CsA. Data are means and SEM from 3 triplicate experiments. (C) Infectivity of unlabeled and CypA-DsRed-containing pseudoviruses. TZM-bl cells were inoculated with equal RT units of viruses in the presence or absence of CsA, and the resulting luciferase activity was measured at 48 h.p.i. Data are means and SD from 5 triplicate experiments. (D) Jurkat CypA+/+ or CypA-/- cells were infected with VSV-G pseudotyped viruses containing or lacking CypA-DsRed. The resulting luciferase activity was measured after 48 h. Results are means and SD from 3 duplicate experiments. (E) Pseudotyped viruses co-labeled with IN-sfGFP and CypA-DsRed were bound to coverslips, permeabilized with TX-100 and fixed with PFA. In parallel samples, the initial colocalization of IN-sfGFP, CypA-DsRed and p24 was preserved by PFA fixation prior to permeabilization (left column). Particles were then treated with CsA (5 μM) or DMSO for 2 min, fixed and immunostained for p24/CA. Scale bars in all panels are 5 μm.

Supporting the notion that oligomerization increases the binding avidity of CypA to Gag, co-localization of CypA-DsRed with IN-sfGFP was on order of magnitude higher than that monomeric CypA-mCherry or CypA-mRFP (Fig 1A and S1A Fig). Western blotting confirmed that CypA-DsRed is abundantly present in virions, but does not affect virus budding or maturation (S2B Fig). Interestingly, overexpression of CypA in producer cells does not improve its incorporation into virions compared to endogenously expressed CypA, in agreement with a defined ~1:10 stoichiometry of CypA/Gag reported previously [23]. In contrast, CypA-DsRed is present in virions in approximately 5-fold excess compared to CypA-mCherry (S2C Fig). The immunoblotting results are consistent with the analyses of IN-sfGFP-associated fluorescence signals suggesting a 4-fold better incorporation of CypA-DsRed over CypA-mCherry (S1B Fig). Based on these results, the apparent CypA-DsRed/Gag stoichiometry in our preparation can be estimated to be in the order of ~1:2.

The specificity of CypA-DsRed incorporation into virions was probed using viruses containing either CA (G89V) or CypA (H126Q) mutants that abrogate the interaction between these proteins [18,23]. Viruses containing the CA G89V mutant failed to efficiently incorporate detectable amounts of CypA-DsRed and, conversely, CypA-H126Q-DsRed was virtually absent in virions containing wild-type (WT) CA (Fig 1A).

We next examined the retention of tagged CypA constructs by virions following a mild permeabilization of coverslip-adhered viruses with saponin, which forms small pores in the viral membrane [27]. Whereas nearly all virus-incorporated CypA-mRFP leaked instantly after saponin treatment, CypA-DsRed was retained (S1D and S1E Fig). The above results show that oligomeric CypA-DsRed: (1) is efficiently and specifically incorporated into HIV-1 particles by tightly binding to the CA region of the Gag polyprotein; and (2) is retained by the viral cores when the integrity of the viral membrane is compromised.

CypA-DsRed incorporation did not significantly affect the fusion-competence or specific infectivity of particles pseudotyped with the Vesicular Stomatitis Virus G protein (VSV-G, Fig 1B and 1C). Parallel samples were inoculated in the presence of the immune-suppressive drug CsA, which binds CypA with high affinity and blocks its interactions with CA [18,28,29]. In agreement with the previously published studies [30,31], CsA did not significantly affect the virus infectivity in HeLa-derived TZM-bl cells (Fig 1C).

We then asked whether CypA-DsRed could also remain associated with HIV-1 cores entering the target cells and thus modulate virus infectivity. Toward this goal, the effect of CypA-DsRed on Jurkat cells infection, which is known to depend on endogenously expressed CypA [30,31], was evaluated. Since the virus-incorporated CypA (from producer cells) does not play a significant role in infection of Jurkat cells [30,31], we hypothesized that this lack of effect is due to a quick loss of monomeric CypA from incoming HIV-1 cores. This hypothesis was tested by measuring the effect of CypA-DsRed incorporation on infection of parental (CypA+/+) and CypA-deficient (CypA-/-) Jurkat cells. Whereas infection of parental cells was not considerably affected by CypA-DsRed, this marker enhanced the infection of cells lacking CypA by 2-3-fold (Fig 1D and S3 Fig). Thus, not only does CypA-DsRed not adversely affect HIV-1 infectivity, but it can functionally complement the lack of CypA expression in target cells. This finding supports the notion that CypA-DsRed tightly binds to HIV-1 CA and is retained by the viral cores released into the target cells.

Having established that virus-incorporated CypA-DsRed does not adversely affect the HIV-1 infectivity, we asked whether over-expression of this marker in target cells can modulate infection. Transient expression of CypA-DsRed in target 293T cells did not significantly affect infectivity of unlabeled VSV-G pseudotyped particles (S4 Fig). In contrast, expression of the HIV-1 restriction factor TRIMCyp [32] markedly reduced the infectivity and this restriction was rescued by CsA treatment (S4 Fig).

### Release of CypA-DsRed correlates with the loss of CA/p24 from HIV-1 cores *in vitro*

To further assess the stability of CypA-DsRed interactions with mature HIV-1 capsids, viruses were adhered to coverslips, permeabilized with TX-100 in the presence of CsA or DMSO (vehicle control), fixed and immunostained for CA/p24. Co-labeling the particles with IN-sfGFP, which is a part of the nucleoprotein complex contained within the capsid, provided a stable reference signal for particles loosing CA/p24. Following the permeabilization, most CypA-DsRed puncta remained colocalized with IN-sfGFP for the duration of incubation with DMSO at room temperature (Fig 1E). CsA treatment of permeabilized particles rapidly displaced CypA-DsRed from IN-sfGFP puncta, markedly reducing the extent of colocalization without considerably affecting the p24 signal (Fig 1E). CsA-mediated displacement of CypA-DsRed from the permeabilized viral particles occurred immediately after addition of the drug (S5 Fig and S1 Movie).

**Fig 2:**
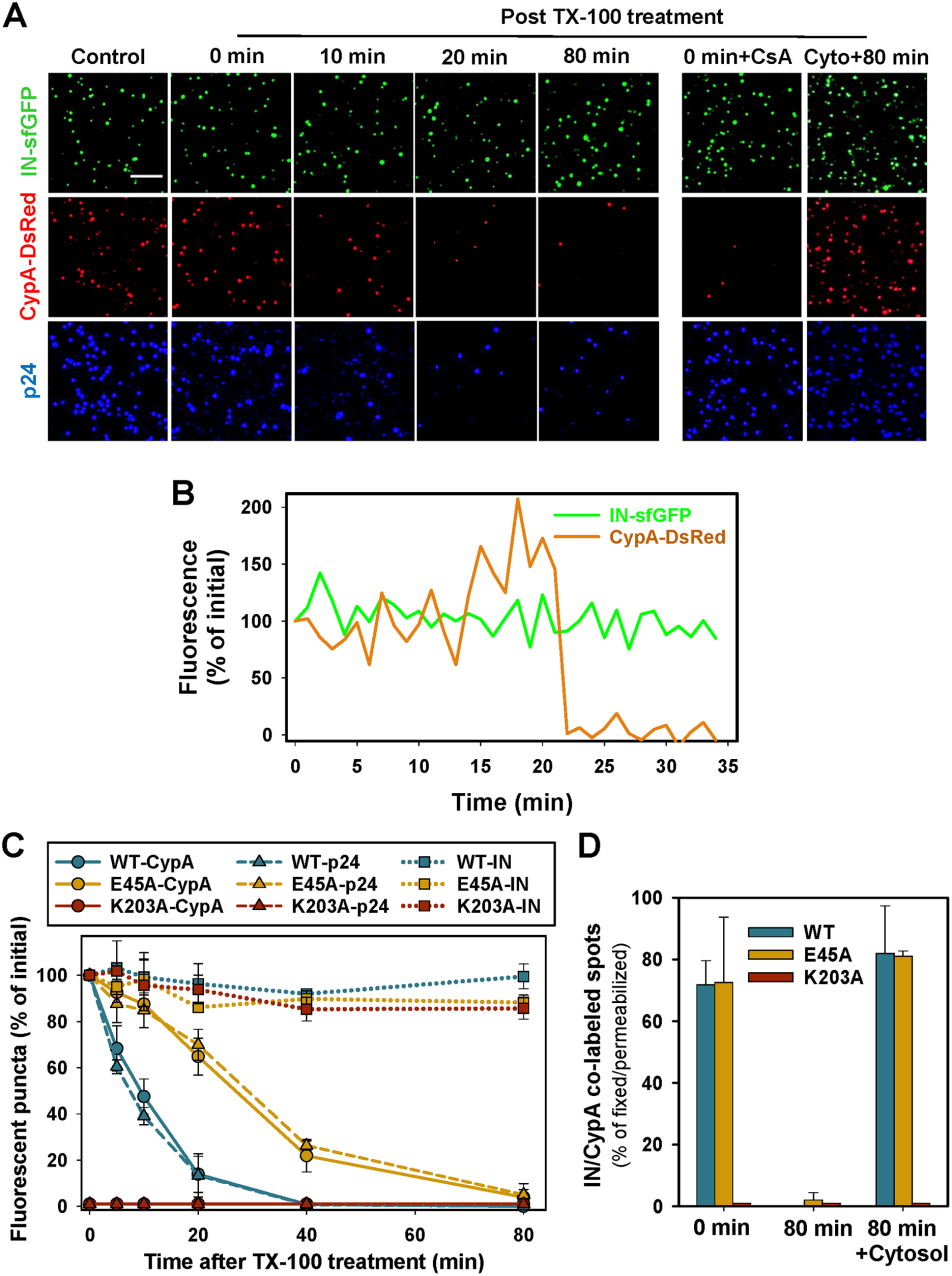
CypA-DsRed dissociation from permeabilized HIV-1 cores is concurrent with the loss of CA and is inhibited by cytosolic extract. (A) IN-sfGFP/CypA-DsRed-labeled viruses were adhered to coverslips, permeabilized as above, fixed with PFA after varied times at 37°C and immunostained for CA/p24. Control viruses (leftmost column) were fixed prior to permeabilization. The two rightmost columns show the effects of CsA (5 μM) and the cytosolic extract from TZM-bl cells. Scale bar 5 μm. (B) Spontaneous release of CypA-DsRed from IN-sfGFP labeled permeabilized viral cores incubated at 37°C without fixation. Sum fluorescence profiles were obtained by single particle tracking and plotted as a function of time TX-100 permeabilization. (C) The average ratios of CypA-DsRed, p24 and IN-sfGFP puncta per image field were determined and normalized to that immediately after permeabilization of pseudoviruses containing WT and the mutant E45A and K203A CA. Four fields of view were imaged for each time point. Data are means and SD from 3 independent experiments. (D) Effect of cytosolic extract on the loss of p24 and CypA-DsRed from the cores bearing the mutant and WT capsids. TX-100-permeabilized viruses were incubated for 80 min at 37°C in the presence or absence of 25 ng/μl cytosol prepared from TZM-bl cells. Results are normalized to samples fixed immediately after permeabilization.

Biochemical studies have shown that detergent-treated purified HIV-1 capsids tend to disassemble in a temperature-dependent manner and that point mutations in CA can strongly modulate the core stability [5]. We thus asked whether the CypA-DsRed loss form immobilized pseudoviruses after permeabilization is accompanied by loss of CA/p24 at 37°C. Co-labeled pseudoviruses were adhered to coverslips, permeabilized and fixed after varied incubation times. The loss of CA from IN-labeled spots was assessed by immunostaining with anti-p24 antibodies. After permeabilization, the CypA-DsRed foci disappeared with a half-time of ~10 min and were lost by 80 min at 37°C, whereas the number of IN-sfGFP puncta remained virtually constant (Fig 2A and 2C and S2 Movie). Once initiated, the release of CypA-DsRed from individual cores was completed in less than 1 min (faster than the temporal resolution of these imaging experiments, Fig 2B). Importantly, p24 and CypA-DsRed puncta disappeared at the same rate (Fig 2A and 2C), implying that the CypA-based marker was released from the viral cores along with the capsid protein.

To ascertain that the release of CypA-DsRed correlates with the HIV-1 core stability, pseudoviruses containing the hyperstable E45A or the unstable K203A CA mutant [5] were prepared and imaged. CypA-DsRed was lost from the permeabilized E45A particles much slower (half-time ~25 min) than from WT CA-containing particles, whereas virtually all CypA puncta disappeared immediately after permeabilization of the K203A viruses (Fig 2C). As with the WT CA, the rates of p24 loss from IN-sfGFP puncta for the two CA mutants were virtually superimposable with the rates of CypA-DsRed loss (Fig 2C). The concomitant release of CypA-DsRed and p24 from permeabilized viral cores and correlation between the core stability and the rate of CypA-DsRed loss strongly suggest that CypA-DsRed is released as a result of CA dissociation from the IN-sfGFP-labeled cores.

Since a human cytosol extract has been reported to stabilize the *in vitro* assembled CA-nucleocapsid complexes [33,34], we asked whether this effect could be observed with *bona fide* HIV-1 cores. A cytosolic extract from TZM-bl cells markedly inhibited of both p24 and CypA-DsRed loss from the IN-sfGFP puncta (Fig 2A and 2D). This important finding shows that human cytosol potently stabilizes the cores obtained by permeabilization of HIV-1 particles that otherwise tend to uncoat under physiological salt and pH conditions.

### The majority of HIV-1 cores lose CypA-DsRed shortly after viral fusion

**Fig 3:**
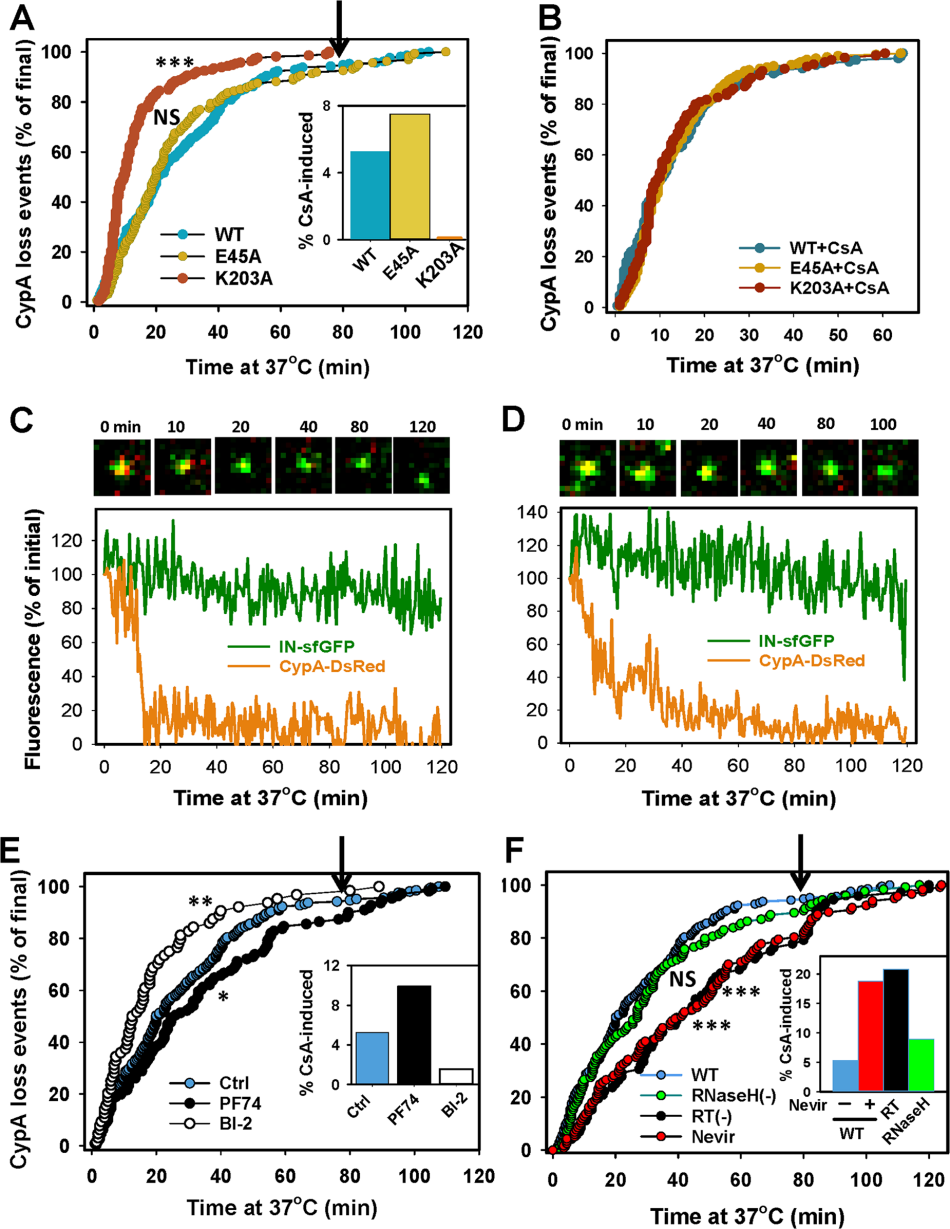
Loss of CypA-DsRed following virus-cell fusion. VSV-G pseudotyped HIV-1 particles bearing WT CA or CA mutants and co-labeled with IN-sfGFP and CypA-DsRed were used to infect TZM-bl cells (~10 pg of p24 per 5·10^4^ cells, MOI 0.008). Viruses were pre-bound to cells in the cold and virus entry/fusion was initiated by adding a pre-warmed buffer. Time-lapse images of a single field of view were taken every 30 sec for 80 min, at which point, 10 μM CsA was added and image acquisition continued for 40 min. (A) Kinetics of CypA-DsRed loss from IN-sfGFP puncta was measured for WT CA and each of the CA mutant viruses in 5 independent experiments. *Inset*: The fraction of double-labeled cores that lose CypA-DsRed in response to 10 μM CsA addition at 80 min (arrow). (B) Same as in panel A, but CsA was added just before starting the image acquisition (0 min) to measure the fusion kinetics. (C, D) Selected images and corresponding CypA-DsRed and IN-sfGFP intensity profiles obtained by single particle tracking. The tracks and images illustrate the relatively quick (C) and the less frequently occurring slow (D) release of CypA-DsRed. Scale bar 0.5 μm. (E) Effects of CA-binding compounds PF74 (10 μM) and BI-2 (20 μM) on the kinetics of CypA-DsRed loss from single post-fusion cores. (F) Reverse transcription accelerates the loss of CypA-DsRed from IN-sfGFP labeled particles. Inhibition of viral DNA synthesis by Nevirapine (10 μM) or by the RT D185N mutation, delayed the kinetics of CypA-DsRed disappearance after fusion. The E478Q mutation, which abolishes RNase H activity, did not exert a significant effect on the CypA-DsRed retention time. *Insets to panels E and F*: The fraction of double-labeled cores that lose CypA-DsRed in response to 10 μM CsA added at 80 min (arrows). Statistical significance was determined by the Mann-Whitney Rank-Sum test.

We next analyzed the dissociation of CypA-DsRed from the HIV-1 cores following their release into the cytoplasm as a result of viral fusion. Viral entry/fusion was synchronized by pre-binding viruses to cells in the cold and quickly raising the temperature to 37°C. Live cell imaging revealed that CypA-DsRed was lost from single IN-sfGFP puncta with a half-time ~23 min after the initiation of virus entry (Fig 3A). This time includes the virus uptake and fusion with acidic endosomes followed by dissociation of the CypA marker from the core. To dissect the virus fusion/uncoating steps, we took advantage of the ability of CsA to quickly displace CypA-DsRed from HIV-1 cores (S5 Fig). The loss of CypA-DsRed in the presence of CsA added at the time of initiating virus entry (0 min) should thus reflect the kinetics of single virus fusion, more specifically, the time of formation of a small fusion pore through which this marker is released into the cytoplasm. Indeed, the kinetics of CypA-DsRed loss from IN-sfGFP particles was considerably faster (half-time ~10 min) in the presence than in the absence of CsA (compare Fig 3A and 3B). These findings reveal a relatively short life span of intact post-fusion HIV-1 cores, which lost CypA-DsRed with half-time of ~13 min (23 min– 10 min). This apparent instability is in stark contrast with the stable association of CypA-DsRed with viral cores at 37°C in the presence of human cytosol *in vitro* (Fig 2A and 2D). It is thus possible that, in agreement with the recent reports [35–37], functional cytoskeleton/molecular motors or other host factors absent in cytosolic extracts drive HIV-1 uncoating in living cells.

Single particle tracking showed that CypA-DsRed was frequently lost in one relatively quick step, typically lasting ~2 min (Fig 3C and S3 Movie), which is similar to *in vitro* uncoating profiles (Fig 2B). A much less frequent gradual loss of the CypA signal (Fig 3D and S4 Movie) was also detected. The quick decay of the CypA signal from individual puncta likely reflects rapid disassembly of the HIV-1 capsid after fusion. The dramatic inhibition of CypA-DsRed loss from permeabilized particles *in vitro* in the presence of cytosol (Fig 2A and 2D) argues against the alternative possibility that CypA somehow dissociates from intact post-fusion cores without the loss of CA.

The relationship between the CypA-DsRed release and HIV-1 capsid stability in the cytoplasm was further examined using the hyperstable (E45A) and unstable (K203A) CA mutants. Whereas the E45A and WT cores lost CypA-DsRed at similar rates, this process was approximately twice as rapid for the K203A cores (Fig 3A). This pattern is somewhat similar to the kinetics of CypA-DsRed loss from permeabilized particles *in vitro* (Fig 2C). In control experiments performed in the presence of CsA to displace the intraviral CypA-DsRed, the rates of viral fusion were identical for WT and the two mutants (Fig 3B). The correlation between the CypA-DsRed retention time and capsid stability implies that this marker is lost due to virus uncoating after fusion. Taken together, the above results establish correlation between the capsid stability and CypA-DsRed retention by native cores *in vitro* and by post-fusion cores in living cells, thus supporting the notion that CypA-DsRed loss reflects HIV-1 uncoating.

The CypA-DsRed release from WT and mutant capsids approached plateau by 80 min at 37°C (Fig 3A). To determine whether any post-fusion cores retained CypA-DsRed at this time point, CsA was added, and imaging was continued for 40 min. CsA displaced CypA-DsRed from an additional 5% and 7.5% of the WT and E45A post-fusion cores, respectively, demonstrating that a small fraction of cores remained intact at that time point (Fig 3A, *Inset* and S5 Movie). By contrast, none of the double-labeled K203A cores responded to CsA treatment, as all CypA signal from post-fusion cores was lost by 80 min (Fig 3A). Thus, the majority of HIV-1 cores lose CypA-DsRed (uncoat) shortly after fusion, but a small fraction (~5%) of cores retains CypA-DsRed for longer time.

We also examined the effects of the capsid-targeting small molecule HIV-1 inhibitors PF74 and BI-2, which have been reported to modulate the core stability [38–41]. The loss of CypA-DsRed from IN-sfGFP puncta was accelerated in the presence of BI-2 (Fig 3E), in agreement with the published biochemical data [33,38]. By contrast, PF74, which appears to alter the stability of the viral core in a concentration-dependent manner [33,38,42–44], modestly delayed the CypA-DsRed loss at 10 μM (Fig 3E).

### Initiation of reverse transcription facilitates loss of CypA-DsRed from post-fusion cores

Several studies have established a link between reverse transcription and capsid uncoating [8,45,46]. We therefore examined the effect of reverse transcription on the apparent stability of post-fusion HIV-1 cores measured by the loss of CypA-DsRed. The RT inhibitor Nevirapine delayed the loss of CypA-DsRed from IN-sfGFP labeled cores compared to the untreated control (Fig 3F). As a result, 17% of the post-fusion cores remained intact (retained CypA-DsRed) by 80 min post-infection in the presence of Nevirapine—a more than 3-fold increase relative to untreated viruses (Fig 3F, *Inset*). In contrast to the observed effect of Nevirapine on early loss of CypA-DsRed, viral DNA synthesis is completed at much later times after infection ([45,47] and S6A Fig). To investigate the effect of early steps in reverse transcription on HIV-1 uncoating, we examined the kinetics of CypA-DsRed loss from post-fusion cores containing the inactive RT D185N mutant, which is unable to synthesize viral cDNA [48], or the E478Q mutant that can initiate viral cDNA synthesis but unable to proceed through the first strand transfer step [49,50] as a result of a mutation in the RNase H pocket [51]. In good agreement with the Nevirapine data, the RT-inactivating D185N mutation delayed the loss of CypA-DsRed (Fig 3F). However, the kinetics of CypA-DsRed loss from virions lacking the RNase H activity was not significantly different from the WT virus (Fig 3F). Collectively, these results suggest that the initiation of reverse transcription is sufficient to destabilize the HIV-1 core at early times post-fusion.

### A minor fraction of post-fusion cores retain CypA-DsRed for a few hours prior to uncoating

**Fig 4:**
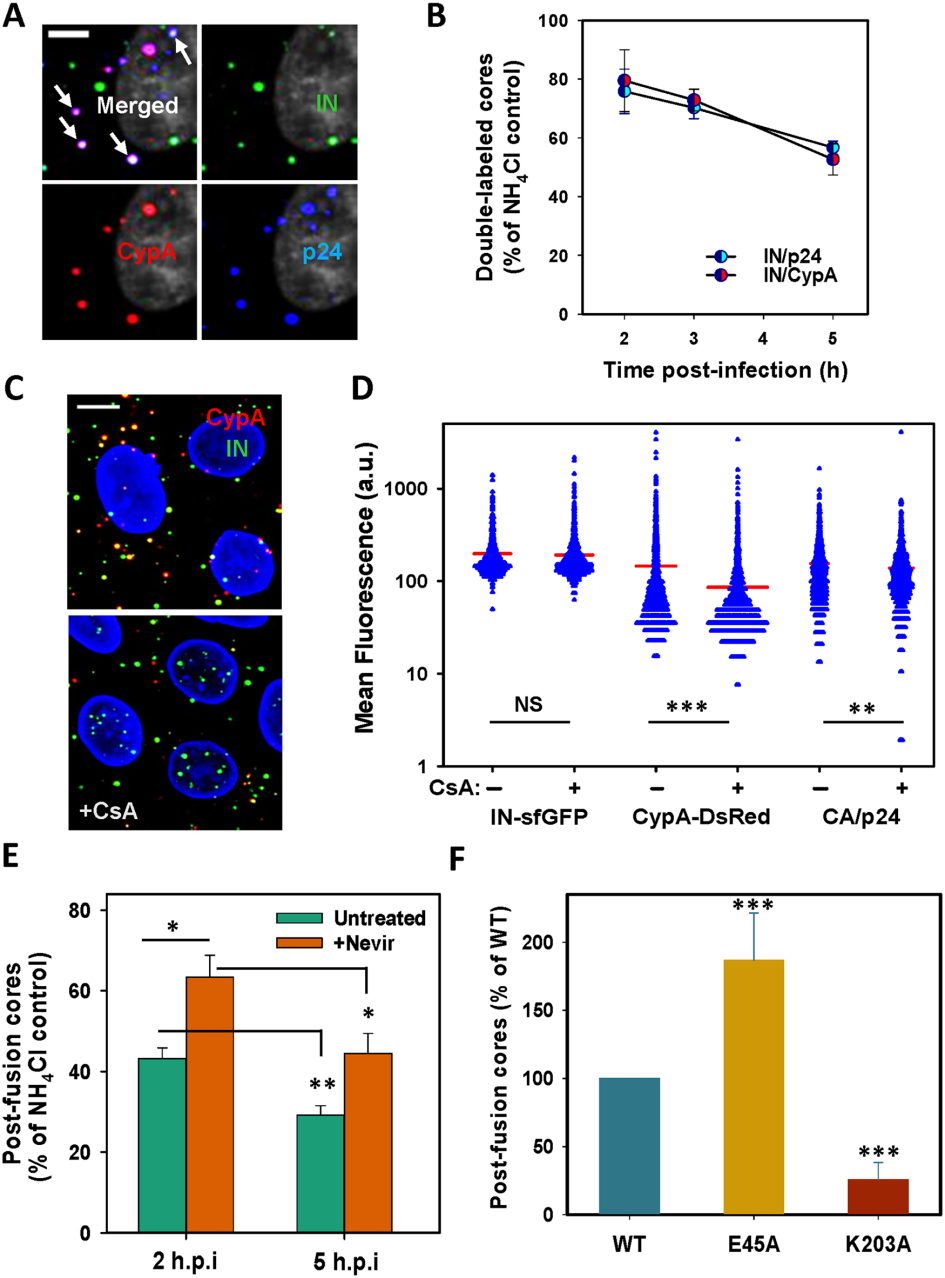
Tight association of CypA-DsRed with post fusion HIV-1 cores. (A) Approximately 100,000 TZM-bl cells were inoculated with ~1 ng (MOI 0.4) of IN-sfGFP (green) and CypA-DsRed (red) labeled pseudoviruses at 37°C, fixed at 4 h post-infection and immunostained for CA/p24. Cell nucleus stained with Hoechst-33342 is colored gray. Scale bar 5 μm. (B) The fraction of double-labeled IN-sfGFP/CypA-DsRed and IN-sfGFP/p24 puncta in TZM-bl cells infected as in panel A after varied times post-infection normalized to the control experiments in which viral fusion was prevented by 50 mM NH_4_Cl. Data are means and SEM from 4 fields of view. (C) TZM-bl cells were infected with pseudoviruses for 4 h, as in panel A, treated with 10 μM CsA or DMSO for 30 min at 37°C, fixed and imaged. Cell nuclei are colored blue. Scale bar 5 μm. (D) Quantification of CsA-induced CypA-DsRed displacement from post-fusion cores shown in panel C. The mean fluorescence intensity of CypA-DsRed and CA/p24 within IN-sfGFP spots was calculated for control and CsA-treated samples. Data are means and SD from 4 fields of view. (E) The number of intact post-fusion cores at indicated times after infection carried out in the presence of Nevirapine (Nevir, 10 μM) or DMSO. The number of post-fusion cores per cell was calculated as the difference between the number of colocalized IN-CypA spots per cell in control and CsA-treated samples for each time point. Data are normalized to NH_4_Cl control. Results are means and SEM from 4 fields of view. (F) TZM-bl cells were infected with ~1.5 ng p24 of viruses bearing WT CA, and the E45A and K203A mutants for 2 h. The number of post-fusion cores for each sample was determined from 5 independent experiments, as described in panel E, and normalized to number of WT CA.

In order to detect rare post-fusion cores at late times after infection, we inoculated cells with a ~50-fold higher amount of virus (MOI 0.4) compared to that used for single virus imaging in live cells (see Materials and Methods). Under these conditions, a considerable number of IN-sfGFP/CypA-DsRed labeled particles, likely a mixture of endosome-resident viruses and stable post-fusion cores, was observed at late times after infection (Fig 4A). The identical rates of reduction in the fractions of IN puncta containing CypA-DsRed and IN puncta positive for p24 staining (Fig 4B) are consistent with slow uncoating of a fraction of HIV-1 cores. To distinguish between virions trapped in endosomes and stable post-fusion cores, cells were treated with CsA prior to fixation. Intact post-fusion cores, defined as double-labeled particles losing CypA-DsRed in response to CsA treatment (Fig 4C and S6 Movie), were observed as late as 6 h.p.i. The average CypA and p24 signals from the IN-sfGFP spots were significantly reduced after CsA treatment (Fig 4D). Consistent with this result, CsA treatment markedly diminished IN/CypA and p24/CypA colocalization (S7 Fig). The CsA-mediated reduction in the mean p24 signal (Fig 4D) is unexpected and may be indicative of HIV-1 capsid destabilization as a result of CypA-DsRed displacement.

At 2 h.p.i., a large fraction of double-labeled particles lost CypA-DsRed after addition of CsA (Fig 4E), whereas CsA-induced loss of the CypA marker was not detected when experiments were done in the presence of NH_4_Cl (S7 Fig). This result shows that about half of puncta in the cytoplasm at 2 h.p.i. are native post-fusion cores containing CypA-DsRed. As expected, this number dropped between 2 and 5 h.p.i. (Fig 4E). In agreement with the stabilizing effect of Nevirapine on HIV-1 cores early after fusion (Fig 3F), a greater number of stable post-fusion cores was detected in Nevirapine-treated cells between 2 and 5 h.p.i. (Fig 4E). We also observed correlation between long-term retention of CypA-DsRed by post-fusion cores and the capsid stability, using the hyperstable (E45A) and unstable (K203A) CA mutants (Fig 4F). Thus, a small fraction of HIV-1 cores does not lose CypA-DsRed for several hours after viral fusion and appear to remain intact. Retention of CypA-DsRed by post-fusion HIV-1 cores for >6 h.p.i. further confirms that this marker is shed along with CA as a result of uncoating.

### Late HIV-1 uncoating occurs gradually and is promoted by reverse transcription

**Fig 5:**
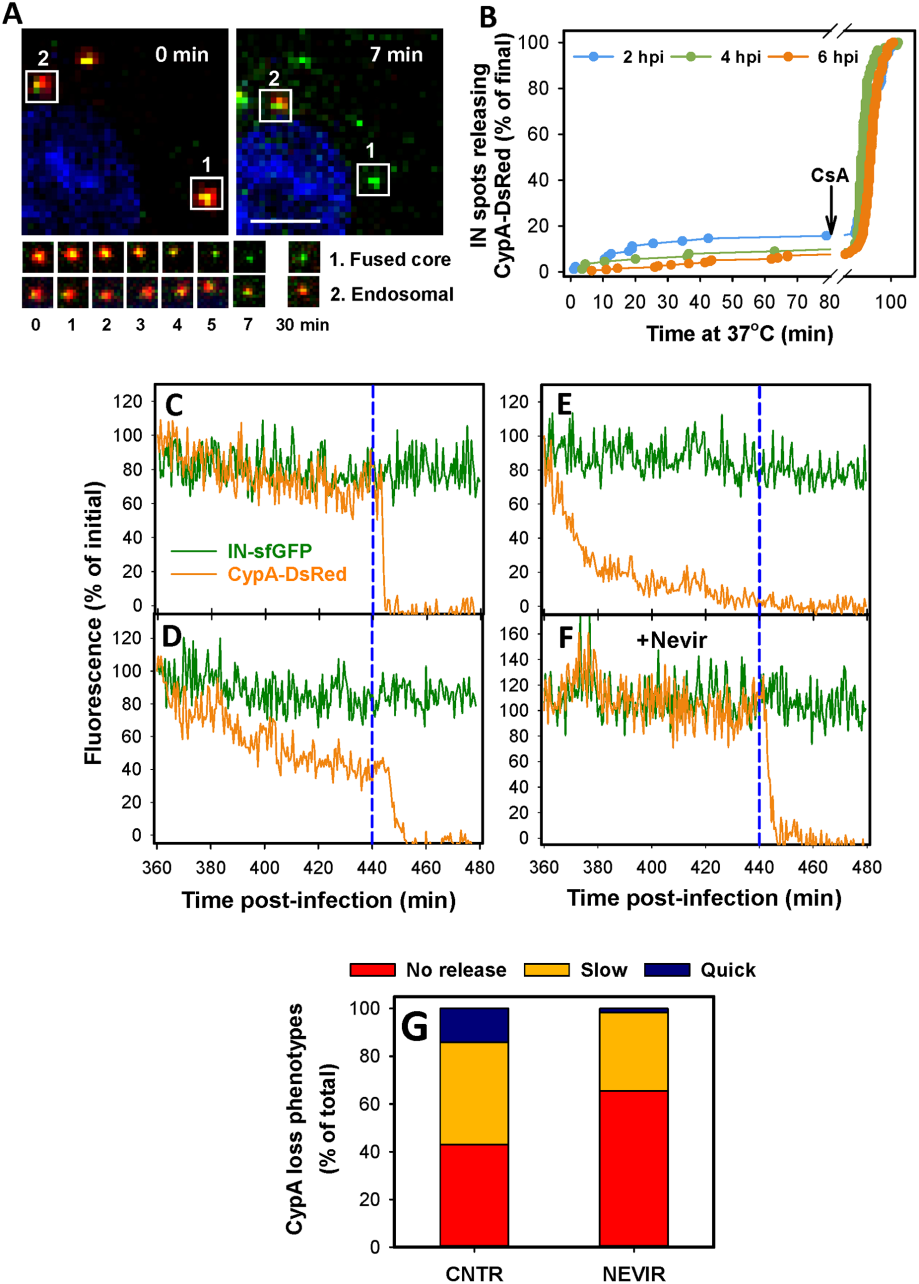
Analyses of spontaneous and CsA-induced release of CypA-DsRed from post-fusion cores. (A) CsA-induces loss of CypA-DsRed from the IN-sfGFP labeled particle 1 in a TZM-bl cell at 4 h.p.i. Particle 2 retained CypA-DsRed. The cell nucleus is colored blue. The lower image panels show blow up views of the two particles at indicated times after CsA addition. Scale bar 10 μm. (B) Kinetics of spontaneous, apparently complete loss of CypA-DsRed from IN-sfGFP labeled cores in TZM-bl cells within 80 min of imaging, starting at 2, 4 or 6 h after infection. CsA (10 μM) was added at 80 min after beginning of image acquisition. (C-G) Single particle tracking analysis of CypA-DsRed loss from IN-sfGFP puncta in TZM-bl cells at 6 h post-infection. Cells were imaged for 80 min, at which point 10 μM CsA was added and imaging continued for 40 min. Examples of a steady level of the CypA marker (C), as well as partial (D) and nearly complete (E) loss of CypADsRed are shown. (F) Typical stable CypA-DsRed and IN-sfGFP signals in the presence of Nevirapine (10 μM). Dashed lines in panels C-F mark the time of CsA addition. (G) Analysis of the single particle tracking results exemplified in panels C-F. The changes in CypA-DsRed intensity within the 80 min imaging window were measured for 142 randomly chosen post-fusion cores in control samples and 122 cores in Nevirapine treated samples. The CypA-DsRed loss was categorized as no release (less than 40% loss of the initial signal), slow (40–70% of the signal) and quick (>70% loss) during the 80 min imaging interval. The fraction of particles that did not release CypA-DsRed within 80 min of imaging increased from 43.0% in control to 65.6% in Nevirapine-treated cells, whereas the slow and quick release events dropped from 42.4% to 32.8% and from 14.6% to 1.6%, respectively.

HIV-1 uncoating at late times after infection was examined by tracking CypA-DsRed/IN-sfGFP- labeled cores in living cells. Time-lapse images were acquired for 80 min, starting at 2, 4 or 6 h.p.i. To identify the pool of post-fusion cores that retained CypA-DsRed by the end of the imaging session, CsA was added at 80 min, and imaging was continued for additional 40 min. CsA displaced CypA-DsRed from the post-fusion cores, but not from unfused virions (Fig 5A). Within each 80 min observation window, only 7–16% of post-fusion cores (depending on the time post-infection) lost the entire CypA-DsRed signal to the background level (Fig 5B). This initial analysis did not include partial loss of the CA marker observed for a significant fraction of particles exhibiting a gradual loss of CypA-DsRed (see Fig 5D and 5E).

Single particle tracking revealed that a large fraction of cores did not exhibit significant loss of CypA-DsRed prior to CsA addition (referred to as “no release”, Fig 5C), while those that lost the marker could be categorized into two types–“slow” and “quick” release (Fig 5D and 5E). The quick release was defined as >70% loss of the CypA-DsRed signal within 80 min (Fig 5E), while slow release corresponded to a drop in the CypA signal down to 30%-60% of the initial value (Fig 5D). In contrast, the levels of IN-sfGFP and CypA-DsRed signals remained steady in control (not fused) particles before and after addition of CsA (S8 Fig).

The relative weights of no release, slow and quick CypA-DsRed release events for randomly analyzed particles are shown in Fig 5G. To ascertain that the observed loss of CypA-DsRed was due to shedding of CA from post-fusion cores, imaging experiments were done in the presence of Nevirapine, which delayed the CypA-DsRed loss at early and late times after infection (Figs 3A and 4E). Consistent with these effects, a majority of post-fusion cores retained the CypA marker in the presence of Nevirapine (e.g. Fig 5F). Analysis of different CypA-DsRed release profiles in Nevirapine-treated cells confirmed that the inhibition of reverse transcription disfavored the slow loss and virtually abrogated the quick loss of the CypA marker (Fig 5G). Collectively, suppression of CypA-DsRed loss by Nevirapine, along with the remarkably stable signals from most post-fusion particles, strongly imply that CypA-DsRed is shed as a consequence of CA dissociation from RTC/PICs and is thus a reliable marker for HIV-1 uncoating.

**Fig 6:**
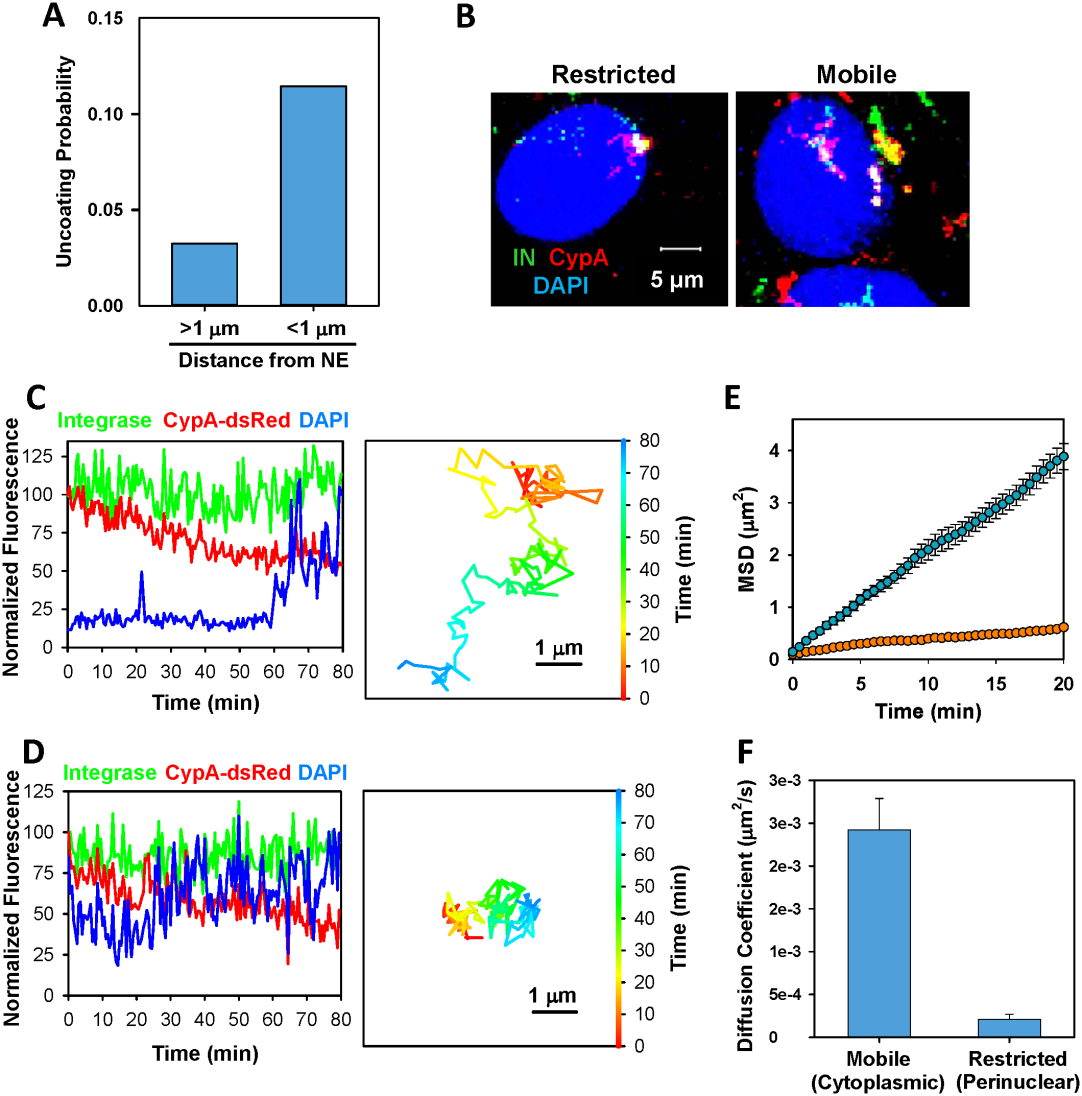
Spatio-temporal analysis of HIV-1 uncoating. (A) A subset of quickly uncoating cores between 4 and 8 h.p.i. (see Fig 5B) was used to determine the particle distance from the nuclear membrane at the time of the apparent complete loss of CypA-DsRed. The number of events away from the nucleus and within 1 μm from the nuclear membrane was normalized to the number of fused cores (the sum of IN spots that lost CypA-DsRed spontaneously and after CsA addition). (B) Images of immobile core at the nuclear membrane (left) and mobile cytoplasmic and perinuclear cores (right). Time-averaged extended projections of 3D images are shown. Nuclei are colored blue. (C, D) Fluorescence intensity profiles and 2D-trajectories of mobile (C) and stationary (D) cores. Track in panel D correspond to the stationary core shown in panel B (see also S7 Movie). Particle trajectories were corrected for the nuclear motion. Blue traces correspond to the nuclear signal overlapping with the tracked core. (E) Mean squared displacement analysis of cores in panels C and D (cyan and orange circles, respectively). (F) Two-dimensional diffusion coefficient calculated from MSD analysis of randomly selected mobile (n = 7) and stationary (n = 11) cores. Error bars are SEM.

We also asked whether the observed uncoating events (loss of CypA-DsRed) exhibited a preference for the cytoplasm vs. the nuclear membrane. To simplify analysis, only a subset of particles exhibiting quick loss of CypA-DsRed (the same subset as in Fig 5B) between 4 and 8 h.pi. were examined. For these events, the distance from the nuclear membrane at the point of complete loss of red signal could be readily measured. The events within 1 μm from the nuclear membrane were categorized as perinuclear, while more distant events were considered cytoplasmic. To determine the probability of uncoating in each category, the number of CypA-DsRed release events was normalized to the respective number of post-fusion cores (those that uncoated in response to CsA) in each category. This analysis showed that the probability of quick release of CypA-DsRed near the nucleus at 4–8 h.p.i. was about 4-fold higher than in the cytoplasm (Fig 6A).

We next asked whether post-fusion HIV-1 cores that uncoated near the nucleus were docked at the nuclear membrane. Only double-labeled post-fusion cores, identified based upon their sensitivity to CsA added at the end of imaging session, were analyzed. Inspection of single particle tracks revealed the existence of the apparently stationary post-fusion cores undergoing restricted movement and relatively mobile cores (Fig 6B). The latter cores were observed both in the cytoplasm and at the nuclear membrane. The cytoplasmic cores typically exhibited relatively quick, sometimes directional motion, as evidenced by their trajectory (Fig 6C) and mean squared displacement (MSD) analyses (Fig 6E, dark cyan circles). By contrast, a fraction of perinuclear cores remained stationary (Fig 6D and 6E, orange circles), consistent with their docking at the nuclear membrane. The 10-fold lower diffusion coefficient of the stationary vs. mobile cores (Fig 6F) further supports that notion that some cores were docked at the nuclear membrane. Analysis of the CypA-DsRed release profiles did not reveal a clear correlation between core uncoating and restricted motion: loss of CypA-DsRed was observed both for mobile and stationary particles (Fig 6C and 6D show slow release of CypA-DsRed). We often observed uncoating initiated in the cytoplasm that continued after particle docking at the nuclear membrane (S9 Fig). To conclude, whereas further experiments and more detailed analyses are needed to delineate the relationship between core mobility and uncoating, our preliminary spatial analysis indicates that quick CypA-DsRed release events from the stable post-fusion cores tend to occur in the vicinity of the nuclear envelope.

## Discussion

In this study, we developed a novel approach to detect the loss of CA from incoming HIV-1 cores in living cells, using the oligomeric CypA-DsRed marker. Previous attempts to label HIV-1 CA with a monomeric fluorescently tagged CypA were unsuccessful [20], most likely because of its low affinity for CA, estimated to be in the micromolar range [22–24,52,53]. In contrast, CypA-DsRed specifically incorporates into virions and remains bound to CA in the context of a mature core, enabling the visualization of single virus entry/uncoating without the need for genetic or pharmacologic manipulations that could perturb multiple functions of the CA protein. Importantly, the CypA-DsRed binding to the core is sufficiently tight to prevent its release after removal of the viral membrane *in vitro* or within several hours after virus fusion with cells. Except when displaced by CsA, CypA-DsRed is lost from IN-labeled RTC/PICs concurrently with loss of CA/p24.

CypA has been implicated in multiple steps of HIV-1 entry, including: (i) CA uncoating [54,55]; (ii) virus restriction by rhesus TRIM5α [56]; (iii) evasion of innate immune sensing [57]; (iv) reverse transcription [58]; and (v) post-nuclear entry events [22,59]. These functions have been attributed to CypA expressed in target cells, whereas the producer cell-expressed CypA that is incorporated into virions has been demonstrated to be inconsequential for infection [30,31]. Remarkably, however, the virus-incorporated CypA-DsRed was able to functionally complement the lack of CypA in the target Jurkat CypA-/- cell line (Fig 1D). This important result supports the notion that, contrary to the virus-incorporated CypA that is quickly shed from post-fusion cores and replaced by CypA in target cells, the oligomeric CypA-DsRed remains tightly associated with the core. Interestingly, unlike the artificial dimeric and trimeric CypA constructs that bind more tightly to CA and inhibit HIV-1 infection when expressed in target cells [25], CypA-DsRed expressed in target 293T cells did not affect HIV-1 infection (S4 Fig). The basis for this difference is currently unclear. We surmise that a long flexible linker between CypA and DsRed allows for multivalent interactions with the core by adapting to the CA lattice rather than disrupting it. Whereas we cannot rule out an effect of CypA-DsRed on the stability of the HIV-1 core, as has been reported for monomeric CypA [33,54,58,60], the lack of a negative effect on HIV-1 infectivity when expressed in producer or target cells, along with the existence of apparently intact post-fusion cores (that shed CypA-DsRed upon CsA addition) in the cytoplasm several hours after synchronized infection, show the utility of CypA-DsRed as a marker for HIV-1 uncoating.

Our conclusion that CypA-DsRed reports the dissociation of CA from the viral core is also strongly supported by the excellent correlation between the HIV-1 capsid stability and loss of this marker, along with p24, from IN-labeled cores *in vitro*. Unlike other reported *in vitro* uncoating assays [5], the CypA-based assay enables the visualization of CA on mature native HIV-1 cores without the need for additional manipulations (e.g., ultracentrifugation) known to destabilize the fragile cores. In agreement with a previous study of HIV-1 core stability [33], we also observed that cytosolic extract from human cells inhibited the loss of CypA-DsRed/CA *in vitro*, thus enabling biochemical approaches to identify host factors that regulate the HIV-1 uncoating. Although the loss of CypA-DsRed from IN-labeled puncta in living cells exhibited more complex phenotypes than in *in vitro* experiments, we were able to demonstrate correlation between loss of this marker and the core stability using CA mutants.

We emphasize that an observed loss of CypA-DsRed should not necessarily be interpreted as complete dissociation of CA. Due to the relatively high background signal in living cells, single virus imaging is only possible with a threshold number of fluorescent protein molecules in these particles. Thus, a small fraction of CA could remain associated with pre-integration complexes, as recently reported in fixed cell imaging studies [38,61,62].

**Fig 7:**
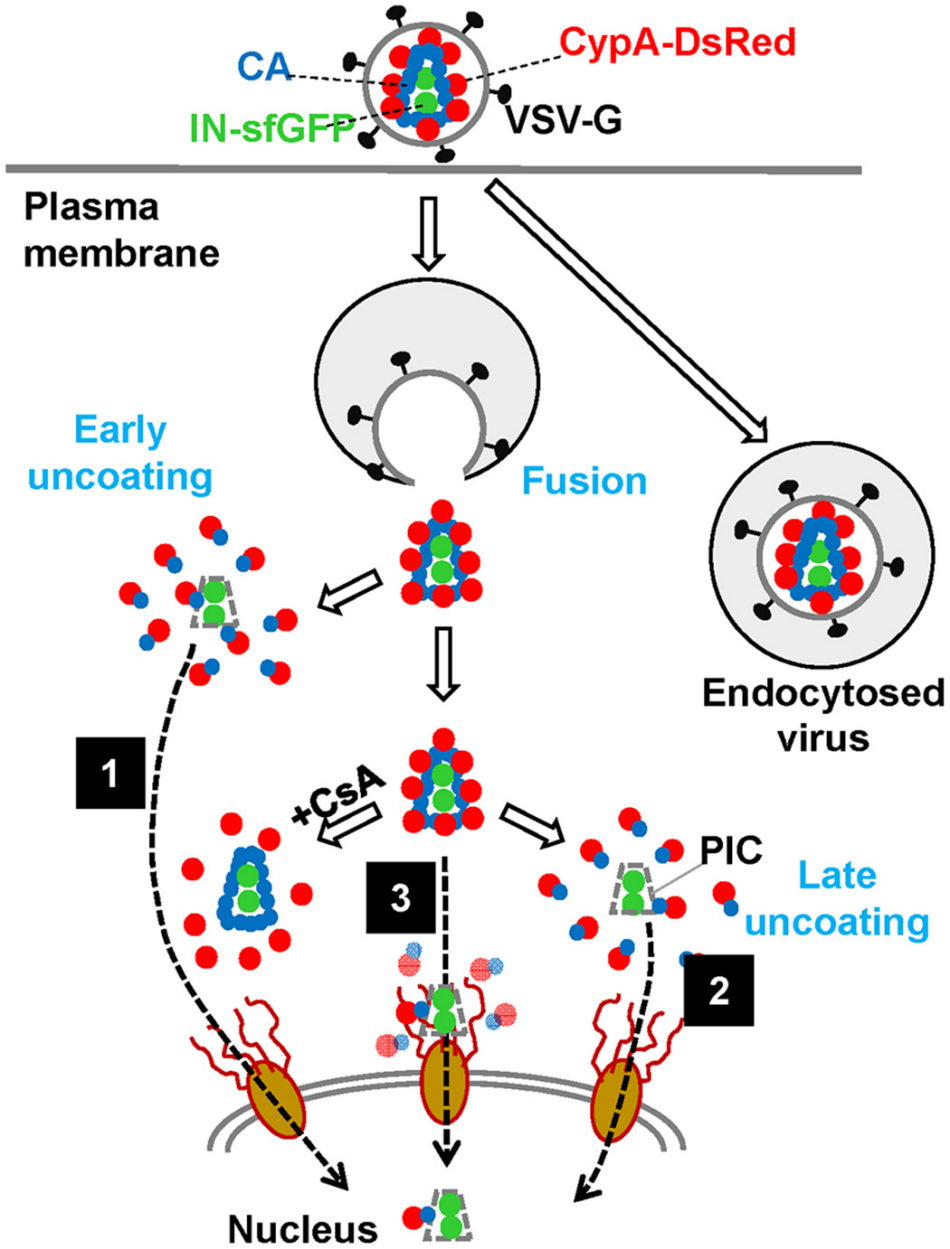
CypA-DsRed enables visualization of HIV-1 CA shedding. CypA-DsRed remains associated with post-fusion cores until CA is shed from IN-sfGFP-labeled cores. Loss of CA/CypA-DsRed occurs either within the first hour after initiating virus entry (pathway 1) or over a period of several hours (pathway 2). An alternative uncoating at the nuclear pore (pathway 3) is also illustrated. Dashed arrows indicate putative nuclear import of pre-integration complexes; these are not meant to relate the sites of uncoating to productive infection.

Three principal models for HIV-1 uncoating in target cells have been proposed (Fig 7, reviewed in [1]). Uncoating may occur: (1) immediately after virus fusion; (2) gradually, during cytoplasmic trafficking of post-fusion cores; and (3) at the nuclear pore. In this study, we observed intracellular particles that appear to represent all three types of uncoating. We found that, surprisingly, the majority of HIV-1 cores lost CypA-DsRed soon after release into the cytoplasm. Since HIV-1 cores are quite stable *in vitro* in the presence of cytosolic extract (Fig 2D), such a dramatic loss of integrity could be caused by innate host factors that induce HIV-1 uncoating and/or by initiation of reverse transcription (Fig 3F), which is unlikely to occur in our *in vitro* experiments. In stark contrast to the rapidly-uncoating cores, a small fraction of particles (<5%) retained CypA-DsRed for several hours after infection. The very slow loss of CypA-DsRed signal from these cores, together with inhibition of CypA-DsRed loss by Nevirapine, supports the gradual uncoating model [8,45]. Finally, a fraction of late-uncoating cores that approached the nuclear membrane exhibited restricted movement (Fig 6B and 6C), which is consistent with HIV-1 uncoating at the nuclear pore [63–65]. Additional single particle tracking experiments using CypA-DsRed, combined with CA mutants exhibiting nuclear entry defects, will be useful for validating this model. Note that our findings apply to HeLa-derived cells that differ from other cell types in their uncoating/infection requirements [45,57].

An important question that remains to be answered is which of the observed uncoating events—early or late—lead to productive infection. There is biochemical and virological evidence supporting early HIV-1 uncoating [8,66]. The functional CsA washout assay [8], which relies on the kinetics of HIV-1 escape from the host restriction factor TRIMCyp, yielded a half-time of ~25–45 min for HIV-1 uncoating in owl monkey cells and ~9 min in HeLa-derived cells [8,45,67]. The latter value is close to our estimate of the half-time of post-fusion HIV-1 uncoating in HeLa-derived cells (~13 min), which is delayed upon inhibition of reverse transcription (Fig 3F). However, since the structural basis for HIV-1 escape from TRIMCyp restriction in the CsA washout assay has not been fully elucidated [1], the rare post-fusion cores which survive for several hours in the cytoplasm may also contribute to productive entry. This notion is supported by the slow kinetics of reverse transcription observed in functional assays ([38] and Fig 6A) and by inhibition of the late CypA-DsRed release events by Nevirapine (Figs 4E and 5G).

In conclusion, we have developed a novel method for elucidating the HIV-1 uncoating process in living cells and *in vitro*, using CypA-DsRed as a marker for CA. This technological advance provides a powerful means to elucidate the spatial and temporal details of HIV-1 uncoating and the role of host factors in regulating this critical step of virus entry.

## Materials and Methods

### Plasmids, cell lines and reagents

CypA was PCR-amplified from genomic DNA and cloned into pmRFP-N1 (Clontech, Mountainview CA). The plasmid pCypA-DsRed and pCypA-mCherry was constructed by replacing the mRFP in CypA-mRFP with respective fluorescent proteins after PCR amplification and cloning between BamHI/NotI sites. CypA was linked to a fluorescent protein through a flexible linker GSGGSGGSGGQSTVPRARDPPVAT. pMDG-VSVG expressing VSV-G glycoprotein, a gift from Dr. J. Young (The Salk Institute for Biological Studies, La Jolla, CA), the plasmid encoding for the pNL4.3 Nef-HA was a gift from Dr. Massimo Pizzato (University of Trento)[11,68]. The Vpr-IN-sfGFP plasmid was a gift from Dr. Anna Cereseto (University of Trento) [11]. The pR9ΔEnv vector containing the WT CA or CA mutations E45A, and K203A were described previously [5]. Mutations encoding the G89V substitution in CA and the E478Q substitution in the RNase H domain of RT were introduced into pR9ΔEnv vector through site-directed mutagenesis. A mutation was introduced by site directed mutagenesis into the pCypA-DsRed plasmid at amino acid position H126 to generate the pCypA-H126Q-DsRed construct. Retroviral vector LNCX2 encoding TRIM-CypA-eCFP was a gift from Dr. Paul Bieniasz [67].

The following reagents were obtained from the NIH AIDS Reference and Reagent Program, Division of AIDS, NIAID, NIH: pNL4-3.Luc.R-E- from Dr. Nathaniel Landau, pMM310 plasmid encoding the BlaM-Vpr was donated by Dr. Michael Miller [69]; TZM-bl cells expressing CD4, CXCR4 and CCR5 were donated by Drs. J.C. Kappes and X. Wu [70]; Jurkat T and Jurkat.T CypA-/- cells were donated by Drs. D. Braaten and J. Luban [71]; anti-p24 antibody AG3.0 was donated by Dr. J. Alan [72]; RT inhibitor Nevirapine; and HIV protease inhibitor Saquinavir; HIV Immunoglobulin (HIV-IG), donated by Dr. Luiz Barbosa.

HEK293T/17 cells were obtained from the ATCC (Manassas, VA). 293T/17 and TZM-bl cells were grown in high glucose Dulbecco's Modified Eagle Medium (DMEM, Mediatech, Manassas VA) with 10% Fetal Bovine Serum (FBS, Sigma, St. Louis, MO) and 100 U/ml penicillin-streptomycin (Gemini Bio-Products, Sacramento, CA). The growth medium for HEK 293T/17 was supplemented with 0.5 mg/ml G418 sulfate (Mediatech, Manassas VA). Jurkat T-cells and Jurkat T-cells CypA-/- were propagated in RPMI-1640 medium.

CsA was obtained from (Calbiochem), dissolved in DMSO at a concentration of 50 mM and stored at -20°C. The HIV-1 CA binding inhibitors, PF74 (PF-3450074) and BI-2, were described previously [39,40]. The CCF4-AM substrate for the BlaM assay, GeneBLAzer *in vivo* Detection Kit was from Invitrogen, Bright-Glo luciferase assay kit was from Promega. Saponin was obtained from Sigma Aldrich.

### Virus production, quantification and western blotting

Fluorescently labeled pseudoviruses were produced and characterized, as described previously [11,68,73]. Briefly, HEK293T/17 cells grown in a 100 mm dish were transfected with the plasmids encoding for the HIV-1 backbone pR9ΔEnv (8 μg), along with VSV-G (1 μg), Vpr-IN-sfGFP (4 μg) and CypA-fluorescent protein plasmids (4 μg) using the JetPrime Transfection reagent (VWR, Radnor, PA). Control viruses were produced without CypA-DsRed. For infectivity assays, pseudoviruses were produced as above but without the Vpr-IN-sfGFP plasmid. Alternatively, NL4-3-based pseudoviruses were produced by co-transfecting VSV-G (1 μg), pNL4.3R-E-Luc (8 μg), Nef-HA (4 μg) and CypA-DsRed (4 μg). Where noted, saquinavir was used at a final concentration of 500 nM to generate immature particles. Twelve hours after transfection, the medium was replaced with 7 ml of fresh DMEM/10% FBS without phenol red, and the sample incubated for additional 36 h at 37°C, 5% CO_2_. Viral supernatant was collected, pooled, filtered through a 0.45 μm filter and quantified for p24 content using AlphaLISA immunoassay kit (PerkinElmer, Waltham, MA) or for the RT activity using the PERT protocol [74]. Viruses were aliquoted and stored at -80°C.

For Western blotting, the viruses were pelleted through a 20% sucrose cushion by centrifuging at 100,000×g for 2 h at 4°C, using the SW41 swinging bucket rotor (Beckman, Indianapolis IN), re-suspended in PBS and lysed with 0.5% Triton X-100 for 30 min at room temperature. Equal amounts of p24 were loaded onto a 12% polyacrylamide gel (Bio-Rad, Hercules, CA). Proteins were transferred onto a nitrocellulose membrane, blocked with PBS/0.1% Tween20/10% Blotting-grade Blocker (Bio-Rad) for 1 h at room temperature and incubated with HIV IG (1∶3000 dilution), rabbit anti-Cyclophilin A antibody (Millipore) (1∶500 dilution), or anti-α-tubulin (Sigma) (1∶3000 dilution) in PBS/0.1% Tween20/5% Blocking-grade Blocker overnight at 4°C. Horseradish peroxidase-conjugated (HRP) goat anti-rabbit antibody (1∶500 dilution, Santa Cruz, Dallas, Texas), HRP-Protein G (1∶2000, Bio-Rad) or HRP-rabbit anti-mouse (Millipore) were employed for protein detection using a chemiluminescence reagent from GE Healthcare. The resulting signal was visualized on the Chem-Doc Imager (Bio-Rad). PrecisionPlus Protein Standards (Kaleidoscope Bio-Rad) were used for molecular weight markers.

### Virus-cell fusion and infectivity assays

Virus fusion activity was measured using the BlaM assay, as described previously [68]. For these experiments, pseudoviruses were produced by transfection with plasmids expressing pR9ΔEnv, VSV-G, BlaM-Vpr, with or without the plasmids expressing CypA-DsRed. Infectivity assays were performed using TZM-bl cells or Jurkat cells. Indicated amounts of p24 or RT units of viral preparations were spinoculated onto cells at 1,500×g, 4°C for 30 min and cells were cultures with or without CsA (5 μM) for 48 h prior to reading the luciferase activity, as described in [75].

### CypA displacement and core-stability measurements *in vitro*

Pseudoviruses co-labeled with IN-sfGFP and CypA-DsRed were allowed to bind to 8-well chambered coverslips or to 96-well black glass-bottom sensoplates (Greiner Bio-One, Monroe, NC) treated with Cell-Tak cell and tissue adhesive (BD Biosciences, San Jose, CA), as per the manufacturer’s instructions. Virions were imaged before and after treatment with 0.002% TX-100 for 30 sec at room temperature. Permeabilized viruses were incubated with CsA (5 μM), or DMSO (vehicle control) for 5 min, fixed with 2% PFA, and immunostained for CA/p24. For core-stability measurements, coverslip bound viruses were permeabilized as above and washed. Samples were further incubated at 37°C in a CO_2_ incubator for 0, 5, 10, 20, 40 or 80 min and fixed with PFA. Control samples were fixed with PFA and then permeabilized with 0.1% TX-100 and subject to immune-labeling with mouse anti-p24 AG3.0 antibody. In parallel samples, TX-100-permeabilized viruses were treated with a cytosolic extract (25 ng/μl) from TZM-bl cells for 80 min and fixed with PFA. Cytosolic extract was obtained by treating 1×10^7^ cells with 200 μl of 10 μg/ml digitonin for 10 min on ice. The supernatant was collected and cleared of cellular debris by centrifugation at 10,000×g for 5 min. Images of 4 randomly chosen fields of view were acquired for each time point.

### Analysis of CypA-DsRed loss from post-fusion cores by fixed cell imaging

For fixed cell analyses, uniform virus binding across the samples was ensured by spinoculating onto coverslip-grown TZM-bl cells placed into a 24-well plate. Briefly, ~100,000 TZM-bl cells were grown on poly-L-lysine coated round coverslips overnight in a 24-well plate and infected by spinoculation at 1500×*g*, 4°C for 20 min with 1–1.5 ng of viral p24 (MOI 0.4–0.6). Cells were washed, exposed to pre-warmed DMEM and incubated at 37°C in a CO_2_ incubator for 2, 4, 5 hours. Cells were then fixed with 2% PFA, permeabilized with 0.1% TX-100 and immunostained for CA/p24 using AG3.0 monoclonal antibody. Parallel infections were performed in the presence of 50 mM NH_4_Cl, with or without 10 μM CsA, for 2 h prior to fixation. To identify intact post-fusion cores, cells were treated with 10 μM CsA for 30 min prior to fixation. When indicated, Nevirapine (10 μM) was added to samples after virus binding. Nuclei were stained with Hoechst-33342 (2 μg/ml) after the fixation step. Images from 4 random fields of view, each containing ~30 cells were collected with a Zeiss LSM780 confocal microscope, and maximum intensity projection images were analyzed. Viral cores co-labeled with IN/CypA and IN/p24 were quantified and plotted as the number of co-labeled cores per cell. The fraction of post-fusion cores was determined by subtracting the number of IN/CypA or p24/CypA colocalized spots after CsA treatment from that in control (untreated) samples for each time point and normalizing to the total number of double-labeled spots detected in the presence of NH_4_Cl.

### CypA-DsRed loss from post-fusion cores in living cells

The CypA-DsRed loss after virus fusion with cells was measured using the VSV-G pseudotyped pR9ΔEnv particles co-labeled with IN-sfGFP and CypA-DsRed. Viruses (10 pg of p24) were bound to 50,000 TZM-bl cells (MOI 0.008) by spinoculation at 1500×*g*, 4°C for 30 min and imaged at 37°C. To examine loss of the CypA marker at late time points post-infection, 50,000 TZM-bl cells were infected with 500 pg p24 (MOI 0.4) of pseudoviruses, as described above. For fixed cell analyses, coverslip-grown 100,000 TZM-bl cells were infected by spinoculation, as above, with 1–1.5 ng of viral p24 (MOI 0.4–0.6). The cells were washed twice, and virus entry was initiated on a pre-heated CO_2_-controlled (5% CO_2_) microscope stage by adding pre-warmed live cell imaging buffer (LCIB, Invitrogen). For live cell experiments, nuclei were stained for 10 min with 2 μg/ml Hoechst-33342 prior to virus binding. CsA (5 μM), BI-2 (20 μM), PF74 (10 μM) or Nevirapine (10 μM) were added to cells decorated with viruses before beginning the image acquisition and maintained throughout the experiment.

### Image acquisition and analyses

3D time-lapse live cell imaging was carried out with a Zeiss LSM780 confocal microscope using a C-Apo 40x/1.2NA water-immersion objective. A suitable field of view was selected, and full cell volume was imaged by acquiring 8–12 Z-stacks spaced by 1 μm every 30 sec, using a minimal power of 405, 488 and 561 nm lasers for Hoechst-33342, sfGFP and DsRed/mRFP/mCherry, respectively. The DefiniteFocus module (Carl Zeiss) was utilized to correct for axial drift. Imaging was done at 37°C using the Zeiss environmental chamber maintained at 5% CO_2_. Acquired image series were converted to maximum intensity projections and analyzed using the ICY image analysis software (icy.bioimageanalysis.org). The complete loss of CypA signal from IN-sfGFP labeled viral particles was blindly annotated by two trained students.

For measuring the CypA and p24 loss from permeabilized virions *in vitro*, single-plane images from 4 independent fields of view were acquired over time with a Zeiss LSM780 microscope using 488, 561 and 633 nm lasers for the green (IN-sfGFP), red (CypA-DsRed) and far-red (p24) channels, respectively. When imaging fixed cells, greater laser powers and line averaging, as well as more stringent sampling in axial direction (typically, ~25 Z-stacks spaced by 0.5 μm), were used. Images were analyzed using the spot detection algorithm of the ICY image analysis software, and the average number of IN, CypA and p24 spots per field of view was determined. For *in vitro* core stability measurements, the average ratios of the number of IN/CypA and IN/p24 spots from 4 fields of view were calculated for the control (fixed and permeabilized), 0 min (permeabilized and immediately fixed), background control (permeabilized, CsA-treated and immediately fixed) and for all time points, as indicated. For each CA mutant, the ratio obtained at 0 min was set to a 100%. At each time point, the ratio obtained for the CsA-treated background control was subtracted. The fraction of cores remaining immediately after TX-100 treatment (0 min) is shown in Fig 2D, where normalization was to the fixed and permeabilized control. Intensity measurements on post-fusion cores at 6-8hrs time points were performed in ImageJ across 3 independent experiments by measuring CypA intensity of single viral puncta at the first time frame and the 80min time frame. Later the percent of CypA-intensity loss in single viral particles were determined for control and nevirapine treated sample in Fig 5G.

Single particle tracking and particle intensity analyses were performed using respective modules available in ICY. Stable cores at the nuclear pore were identified by analyzing the motion of single particles that stayed in the vicinity of the nuclear membrane during the image acquisition. The particle trajectories were corrected for nuclear movement by determining the center of mass for the respective nucleus and tracking its displacement. Two-dimensional diffusion coefficients and MSD were calculated from the particle trajectories using the following equation:

$$MSD\left(\Delta t\right)=\frac{1}{N}\sum_{i=1}^{N}[{\left(x\left(i+\Delta t\right)-x\left(i\right)\right)}^{2}+\left(y\left(i+\Delta t\right)-y\left(i\right){)}^{2}\right]$$

For assessing loss of CypA-DsRed in fixed cells, colocalization of IN-, CypA- and p24-positive puncta was measured using the ICY module. Pairwise colocalization of the three channels within the identified objects was determined. The fraction of post-fusion cores was determined by subtracting the number of IN-sfGFP/CypA-DsRed-labeled particles after CsA treatment from that in untreated control and normalizing to the number of co-labeled puncta in the presence of NH_4_Cl, which blocks VSV-G mediated viral fusion.

### Statistical analysis

Statistical significance was determined by the Mann-Whitney rank-sum test or the Student t-Test, as indicated. p<0.05 (*) was considered significant; p<0.01 and p<0.001 are denoted as ** and ***, respectively.

## Supporting Information

S1 Fig Incorporation of chimeric CypA-fluorescent fusion proteins into HIV-1 particles. (A) Pseudoviruses co-labeled with IN-sfGFP (green) and CypA-DsRed or CypA-mRFP or CypA-mCherry (red) were immobilized on coverslips, fixed, permeabilized with TX-100 and immunostained for CA/p24 (blue). Percent of pair-wise colocalization between the three proteins are shown on the respective image panels. Green numbers show the fraction on IN puncta colocalized with CypA or p24, red numbers are the fraction of CypA puncta colocalized with IN or p24, and blue puncta show colocalization of p24 with the other two proteins. Scale bar 5 μm. (B) Distributions of mean fluorescence intensities of CypA-based core markers (shown in panel A) colocalized with IN-sfGFP puncta. The means and SD from 4 fields of view are shown. Statistical significance evaluated by the Mann-Whitney rank-sum test. (C) Virus producing 293T cells expressing IN-sfGFP and indicated CypA-fluorescent proteins are shown. Scale bar 50 μm. (D) Pseudoviruses co-labeled with IN-sfGFP and CypA-mRFP or CypA-DsRed were adhered to coverslips and subjected to mild permeabilization with saponin (100 μg/ml). Images were acquired immediately before and 5 min after application of saponin. Scale bar 5 μm. (E) Analysis of the CypA-mRFP and CypA-DsRed loss from saponin-permeabilized viruses in panel D. Error bars represent standard error from 3 independent experiments.
(TIFF)

S2 Fig Oligomerization and virus incorporation of fluorescently tagged CypA constructs. (A) Western blot analysis of oligomerization of mRFP, DsRed and CypA fusions with either of fluorescent proteins transiently expressed in 293T cells. Cytosolic extracts were obtained by digitonin treatment as described in Material and Methods. Samples containing 0.25 μg of total protein were boiled for 5 min at 95°C or left at room temperature prior to loading on a 12% PAGE and immunoblot developed using either rabbit anti-mCherry antibody (1:500 dilution, Abcam) or rabbit anti-Cyclophilin A antibody (1:500 dilution, Millipore). (B) Western blot analysis of pseudoviruses produced by transfection of 293T cells with pR8ΔEnv plasmid and either CypA, CypA-DsRed or CypA-mCherry vector. Control CypA-DsRed-labeled samples were produced in the presence of 500 nM HIV-1 protease inhibitor Saquinavir (SQV). Virus samples were purified through 20% sucrose cushion and quantified for p24 content. Equal p24 content containing viral suspension was loaded on a 12% PAGE and immunoblot developed using antibodies against HIV-1 CA, CypA. Lower panels showing CypA expression and loading control tubulin in producer cell lysates. (C) Densitometric quantification of CypA-DsRed and CypA-mCherry incorporation into virions (panel B, top). The intensity of the respective CypA bands was normalized to the total intensity of Pr55 and p24 bands using Image Lab software (Bio-Rad).
(TIFF)

S3 Fig The effect of CypA-DsRed on infection of parental and CypA^-/-^ Jurkat cells. Shown are raw infectivity results for NL4-3/VSV-G pseudoviruses in parental Jurkat cells (A) and in CypA^-/-^ Jurkat cells (B) pertaining to the main Fig 1D. Ten thousand cells were inoculated with 400, 80, or 40 pg of p24 of VSV-G pseudotyped pNLR-E-Luc virus that contained or lacked CypA-DsRed. NL-Cyp1 and NL-Cyp2 denote two different virus preparations containing CypA-DsRed. Luciferase signal was measured at 48 h post infection. Average RLU and SD from duplicate samples of a representative experiment of 4 independent experiments are shown.
(TIFF)

S4 Fig CypA-DsRed expressed in target cells does not restrict HIV-1 infection. 293T cells were transfected with plasmids expressing DsRed, CypA-mRFP and CypA-DsRed, as well as TRIMCyp-eCFP (positive control). Twenty four hours post transfection, the cells were re-plated into 96-well plate, and 16 hours later infected with different dilutions of VSV-G pseudotyped pNL4.3 R-E- Luc virus (based on the RT activity) in the absence (A) or in the presence (B) of 5 μM CsA. Two days after infection, the luciferase signal (RLU) was measured. A representative triplicate experiment from 3 independent experiments is shown. Error bar represents SD. Note the less potent restriction of infection by the TRIMCyp-eCFP fusion protein as compared to unlabeled TRIMCyp reported in the literature (Perez-Caballero et al., *J*. *Virol*. 2005, **79**: 15567–15572). (C) Representative images showing the efficiency of transfection of 293T cells with plasmids expressing the above proteins. Scale bar 50 μm.
(TIFF)

S5 Fig CsA-mediated displacement of CypA-DsRed from IN-sfGFP-labeled permeabilized HIV-1 cores. Same is in Fig 1E, but permeabilized particles were imaged at room temperature without fixation and CsA (5 μM) was added at 75 min. The number of CypA-DsRed puncta is plotted. Error bars are means and SD from 3 image fields. *Inset*: CypA-DsRed release from single permeabilized viral cores upon CsA addition (arrow). Mean fluorescence of IN-sfGFP-colocalized CypA-DsRed obtained by single particle tracking.
(TIFF)

S6 Fig Kinetics of reverse transcription and characterization of RT mutants. (A) Approximately 10,000 TZM-bl cells were infected in a 96 well plate with R9ΔEnv/VSV-G pseudotyped viruses, as described in Materials and Methods. Drugs targeting reverse transcription (Nevirapine, 10 μM) or integration (Raltegravir, 5 μM) were added to wells at indicated time points and maintained throughout the experiment (control cells were infected in the presence of respective amounts of DMSO). The resulting luciferase activity was measured at 48 h.p.i. The average luciferase activity from triplicate samples was measured. Error bars are SD. (B) and (C) Analysis of reverse transcription of RT mutants in target cells. Hela cells (100,000) were inoculated with DNase I-treated VSV-G-pseudotyped R9ΔEnv viruses (100 ng p24). After 2 hr and 8 hr, cells were detached with trypsin, pelleted, and extracts prepared for PCR as previously described (von Schwedler et al., *J*. *Virol*. 1993, 67:4945–4955). qPCR quantification of minus strand strong stop (panel B) and first strand-transfer reverse transcripts (panel C) were performed using stage-specific PCR primers and SYBR green detection. Strong Stop: Forward 5’-GGTCTCTCTGGTTAGACCA-3’; Reverse 5’-AAGCAGTGGGTTCCCTAGTTAG-3’. First Strand Transfer: Forward 5’-AGCAGCTGCTTTTTGCCTGTACT-3’; Reverse 5’-ACACAACAGACGGGCACACAC-3’. Standards consisting of plasmid DNA dilutions were used to generate a standard curve, and copy numbers of test samples determined by interpolation. Controls included cultures of cells inoculated with Env-defective HIV-1 particles lacking VSV-G (Env-) and cultures that were not exposed to virions (No virus). Mean and STD from 2 independent experiments performed in triplicates are shown.
(TIFF)

S7 Fig Release of CypA-DsRed from post-fusion cores by CsA treatment. TZM-bl cells were infected with pR9ΔEnv-based pseudovirus co-labeled with IN-sfGFP and CypA-DsRed. Cells were incubated at 37°C for up to 2 h, at which time CsA (10 μM) was added and incubation continued for an additional 30 min before fixation and immunostaining for CA/p24. (A) Confocal images of the maximum intensity projection of the middle 3 Z-stacks are shown for control (untreated), 10 μM CsA-treated and CsA + 50 mM NH_4_Cl-treated cells. Top panel: CypA-DsRed alone; middle panel: IN-sfGFP+nuclear stain (2μg/ml Hoechst-33322); lower panel: Merged images of IN/CypA/nuclear stain. Scale bar 5 μm. (B) The fraction of IN-CypA, p24-CypA or IN-p24 co-labeled particles in CsA- and DMSO-treated samples is plotted after normalizing to IN-CypA colocalization in the NH_4_Cl+CsA treated control. Error bars represent standard error from 4 imaging fields of view.
(TIFF)

S8 Fig Fluorescence intensities of IN-sfGFP and CypA-DsRed labeled particles residing in endosomes (photobleaching control). Image acquisition started at 6 h.p.i. and CsA was added 80 min after that (arrow). For details, see Fig 5C–5E.
(TIFF)

S9 Fig Loss of CypA-DsRed initiated in the cytoplasm and completed at the nuclear membrane. TZM-bl cells were inoculated with IN-sfGFP/CypA-DsRed labeled particles pseudotyped with VSV-G. Loss of the CA marker started at ~85 min post-infection and reached completion after the core approached the nucleus, as evidenced by the marked increase in the DAPI signal (blue) at ~93 min. Fluorescence signals are normalized to the initial values at 65 min (IN and CypA) or to the final value (DAPI).
(TIFF)

S1 Movie CsA mediates displacement of CypA-DsRed from permeabilized pseudoviruses *in vitro*. **P**seudoviruses co-labeled with IN-sfGFP (green) and CypA-DsRed (red) were bound to glass coverslips and permeabilized with saponin. Addition of CsA quickly displaces CypA-DsRed. Scale bar 5 μm.
(AVI)

S2 Movie Loss of CypA-DsRed from viral cores *in vitro*. IN-sfGFP (green) and CypA-DsRed (red) co-labeled pseudoviruses were bound to glass coverslips, permeabilized with Triton-X100 and incubated at 37°C. Following permeabilization, CypA-DsRed is gradually lost from IN-sfGFP-labeled cores. CypA-DsRed loss is concomitant with loss of p24 (see Fig 2). Scale bar 5 μm.
(AVI)

S3 Movie Quick loss of CypA-DsRed from viral cores shortly after fusion. IN-sfGFP (green) and CypA-DsRed (red) co-labeled pseudoviruses were pre-bound to target cells in the cold and their fusion with cells was initiated by shifting to 37°C at 0 min. *Inset*: Overlay of fluorescence and DIC images. Scale bar 5 μm.
(AVI)

S4 Movie Gradual loss of CypA-DsRed from viral cores after virus-cell fusion. IN-sfGFP (green) and CypA-DsRed (red) co-labeled pseudoviruses were pre-bound to target cells in the cold and fusion was initiated (0 min) by shifting to 37°C. *Inset*: An overlay of fluorescence and DIC images shows that the particle traveled along an extended filopodium toward the center of the target cell prior to gradually loosing CypA-DsRed. Scale bar 10 μm.
(AVI)

S5 Movie CsA-mediated displacement of CypA-DsRed from cores at early time after infection. IN-sfGFP (green) and CypA-DsRed (red) co-labeled pseudoviruses were pre-bound to target cells in the cold and fusion initiated (0 min) by shifting to 37°C. CsA was added 85 min post-infection. *Inset*: An overlay of fluorescence and DIC images. Scale bar 5 μm.
(AVI)

S6 Movie CsA-mediated loss of CypA-DsRed from cores at late times after infection. TZM-bl cells were inoculated with IN-sfGFP (green) and CypA-DsRed (red) co-labeled pseudoviruses for 6 h at 37°C, after which time, the cells were imaged (designated as 0 min). Addition of CsA after 80 min results in a quick loss of CypA-DsRed from long-lived cores. Scale bar 20 μm.
(AVI)

S7 Movie Constrained motion of a post-fusion core at the nuclear membrane. TZM-bl cells were inoculated with pseudoviruses co-labeled with IN-sfGFP (green) and CypA-DsRed (red) and imaged starting at 6 h.p.i. for 80 min at 37°C. An example of an immobile post-fusion core in close proximity to the nuclear membrane (see Fig 6B and 6D). Scale bar 5 μm.
(AVI)
