# Supplementary figures and images for: Time-Resolved Imaging of Single HIV-1 Uncoating In Vitro and in Living Cells
Source: PLoS Pathog. 2016 Jun 20;12(6):e1005709. doi: 10.1371/journal.ppat.1005709 (PMC4913920; doi:10.1371/journal.ppat.1005709)

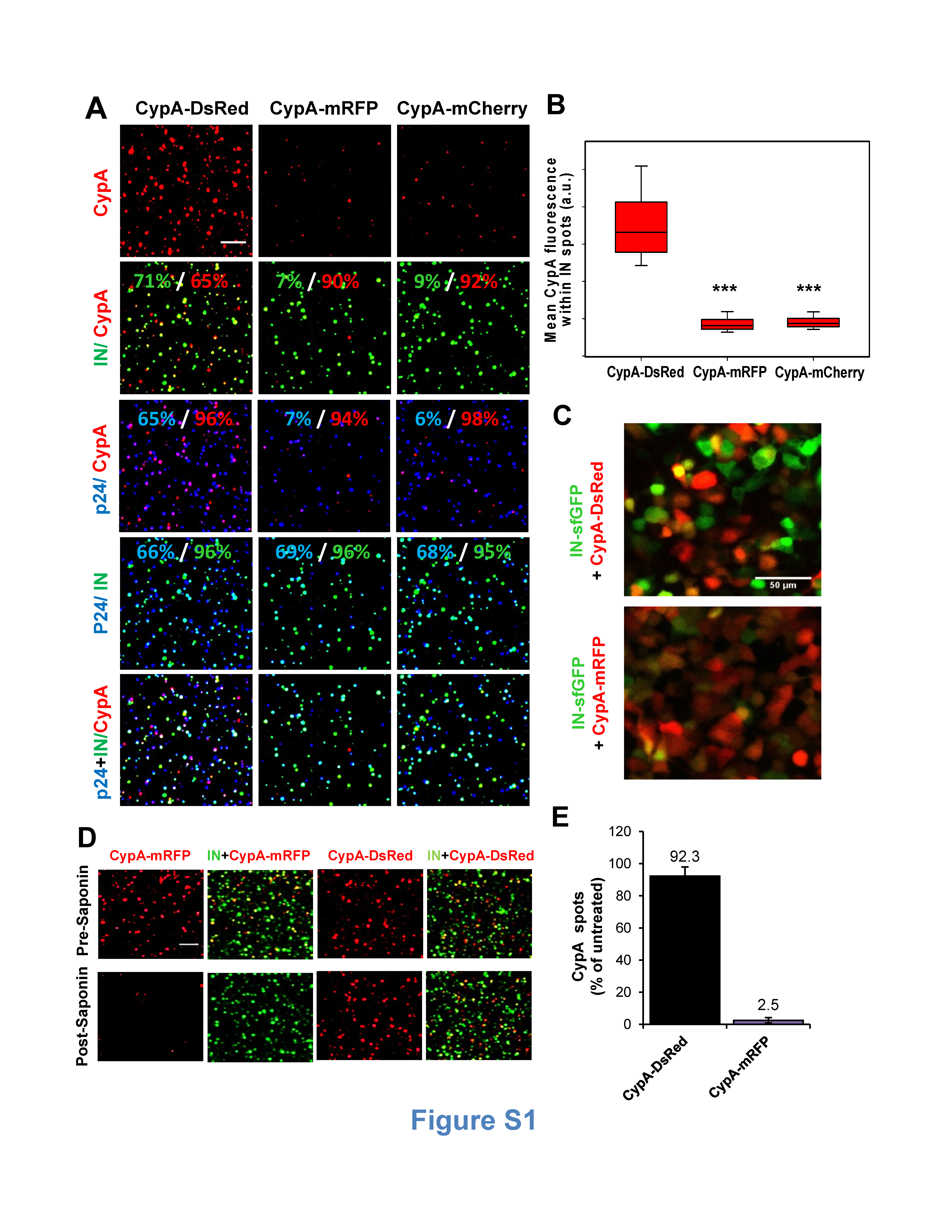

Supplement: S1 Fig — (A) Pseudoviruses co-labeled with IN-sfGFP (green) and CypA-DsRed or CypA-mRFP or CypA-mCherry (red) were immobilized on coverslips, fixed, permeabilized with TX-100 and immunostained for CA/p24 (blue). Percent of pair-wise colocalization between the three proteins are shown on the respective image panels. Green numbers show the fraction on IN puncta colocalized with CypA or p24, red numbers are the fraction of CypA puncta colocalized with IN or p24, and blue puncta show colocalization of p24 with the other two proteins. Scale bar 5 μm. (B) Distributions of mean fluorescence intensities of CypA-based core markers (shown in panel A) colocalized with IN-sfGFP puncta. The means and SD from 4 fields of view are shown. Statistical significance evaluated by the Mann-Whitney rank-sum test. (C) Virus producing 293T cells expressing IN-sfGFP and indicated CypA-fluorescent proteins are shown. Scale bar 50 μm. (D) Pseudoviruses co-labeled with IN-sfGFP and CypA-mRFP or CypA-DsRed were adhered to coverslips and subjected to mild permeabilization with saponin (100 μg/ml). Images were acquired immediately before and 5 min after application of saponin. Scale bar 5 μm. (E) Analysis of the CypA-mRFP and CypA-DsRed loss from saponin-permeabilized viruses in panel D. Error bars represent standard error from 3 independent experiments. (TIFF) [file ppat.1005709.s001.tiff]

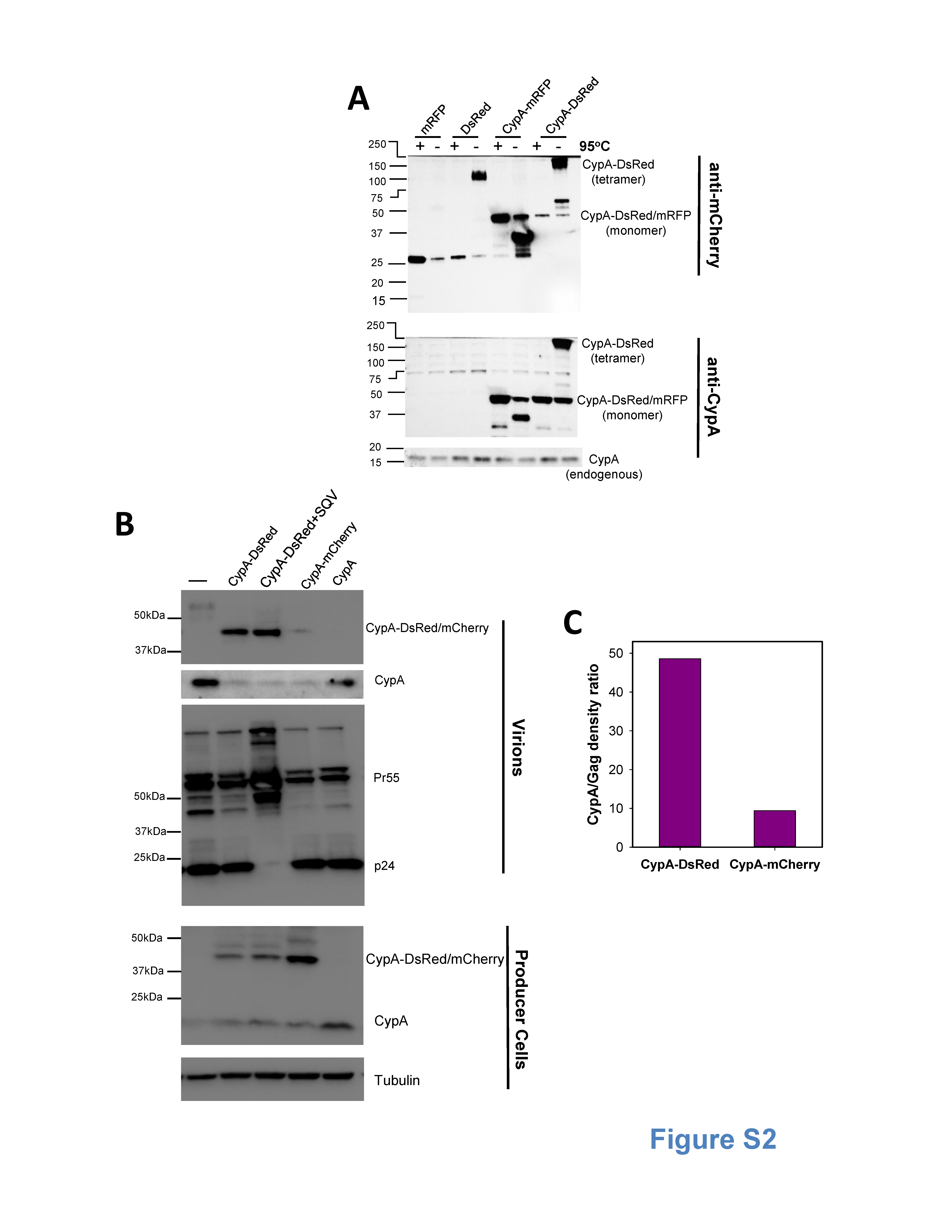

Supplement: S2 Fig — (A) Western blot analysis of oligomerization of mRFP, DsRed and CypA fusions with either of fluorescent proteins transiently expressed in 293T cells. Cytosolic extracts were obtained by digitonin treatment as described in Material and Methods. Samples containing 0.25 μg of total protein were boiled for 5 min at 95°C or left at room temperature prior to loading on a 12% PAGE and immunoblot developed using either rabbit anti-mCherry antibody (1:500 dilution, Abcam) or rabbit anti-Cyclophilin A antibody (1:500 dilution, Millipore). (B) Western blot analysis of pseudoviruses produced by transfection of 293T cells with pR8ΔEnv plasmid and either CypA, CypA-DsRed or CypA-mCherry vector. Control CypA-DsRed-labeled samples were produced in the presence of 500 nM HIV-1 protease inhibitor Saquinavir (SQV). Virus samples were purified through 20% sucrose cushion and quantified for p24 content. Equal p24 content containing viral suspension was loaded on a 12% PAGE and immunoblot developed using antibodies against HIV-1 CA, CypA. Lower panels showing CypA expression and loading control tubulin in producer cell lysates. (C) Densitometric quantification of CypA-DsRed and CypA-mCherry incorporation into virions (panel B, top). The intensity of the respective CypA bands was normalized to the total intensity of Pr55 and p24 bands using Image Lab software (Bio-Rad). (TIFF) [file ppat.1005709.s002.tiff]

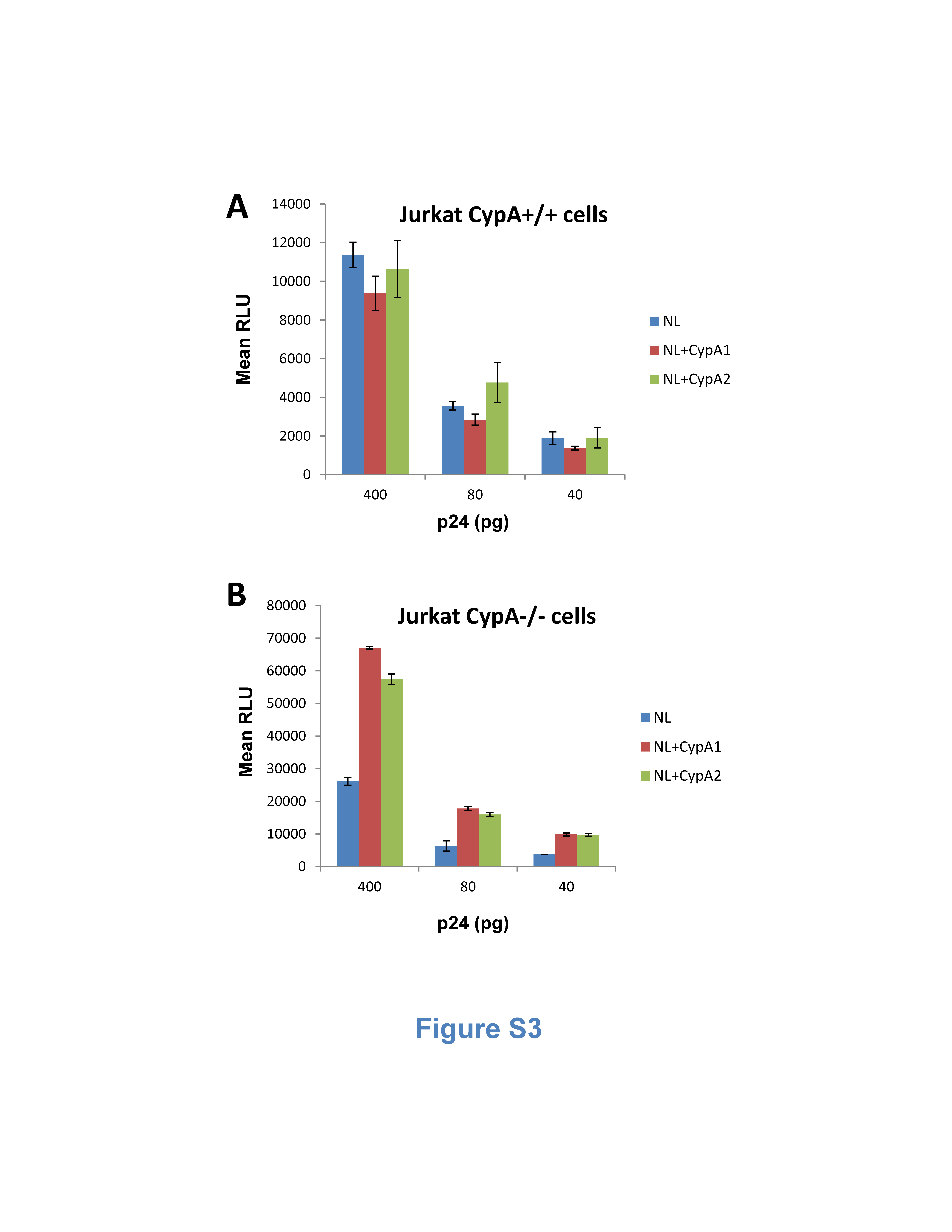

Supplement: S3 Fig — Shown are raw infectivity results for NL4-3/VSV-G pseudoviruses in parental Jurkat cells (A) and in CypA-/- Jurkat cells (B) pertaining to the main Fig 1D. Ten thousand cells were inoculated with 400, 80, or 40 pg of p24 of VSV-G pseudotyped pNLR-E-Luc virus that contained or lacked CypA-DsRed. NL-Cyp1 and NL-Cyp2 denote two different virus preparations containing CypA-DsRed. Luciferase signal was measured at 48 h post infection. Average RLU and SD from duplicate samples of a representative experiment of 4 independent experiments are shown. (TIFF) [file ppat.1005709.s003.tiff]

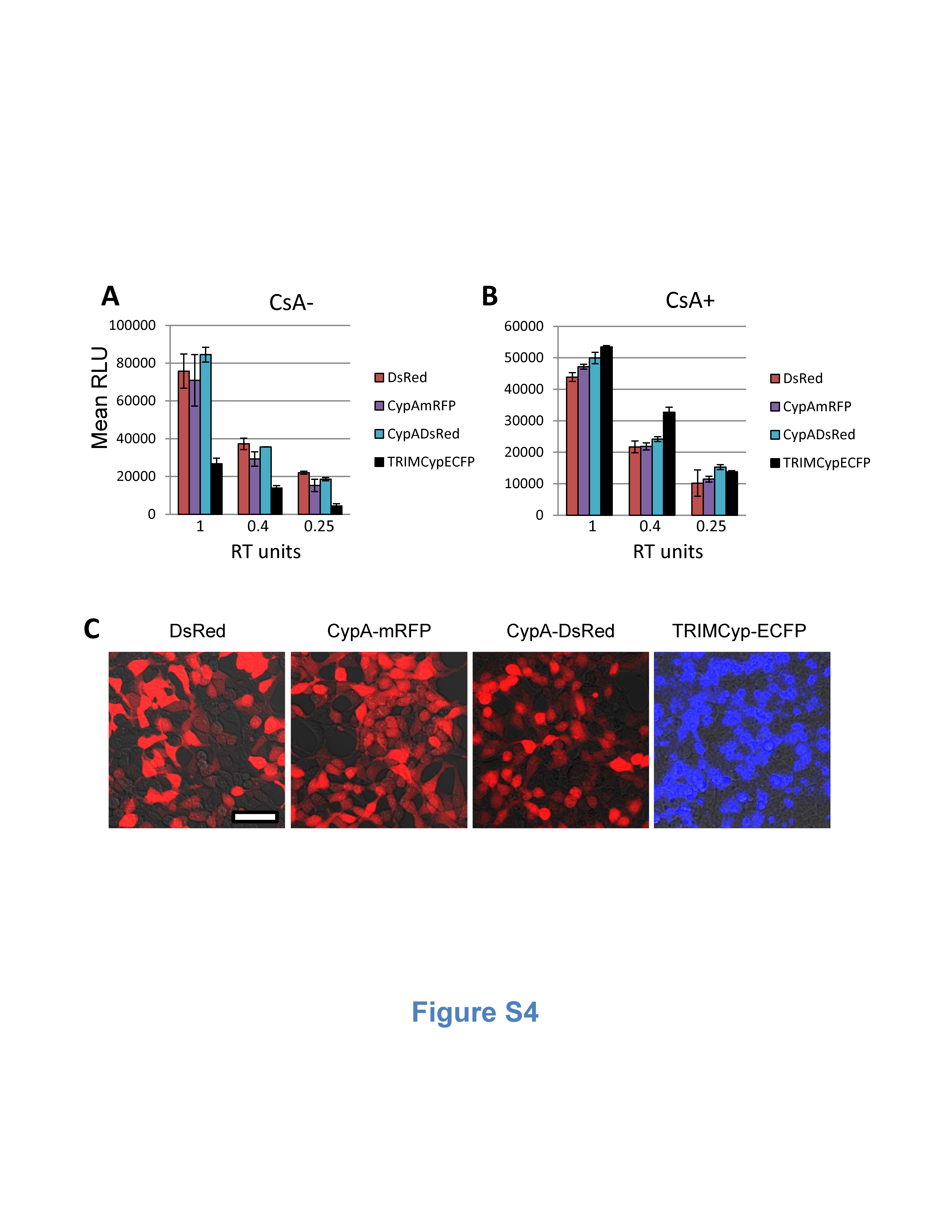

Supplement: S4 Fig — 293T cells were transfected with plasmids expressing DsRed, CypA-mRFP and CypA-DsRed, as well as TRIMCyp-eCFP (positive control). Twenty four hours post transfection, the cells were re-plated into 96-well plate, and 16 hours later infected with different dilutions of VSV-G pseudotyped pNL4.3 R-E- Luc virus (based on the RT activity) in the absence (A) or in the presence (B) of 5 μM CsA. Two days after infection, the luciferase signal (RLU) was measured. A representative triplicate experiment from 3 independent experiments is shown. Error bar represents SD. Note the less potent restriction of infection by the TRIMCyp-eCFP fusion protein as compared to unlabeled TRIMCyp reported in the literature (Perez-Caballero et al., J. Virol. 2005, 79: 15567–15572). (C) Representative images showing the efficiency of transfection of 293T cells with plasmids expressing the above proteins. Scale bar 50 μm. (TIFF) [file ppat.1005709.s004.tiff]

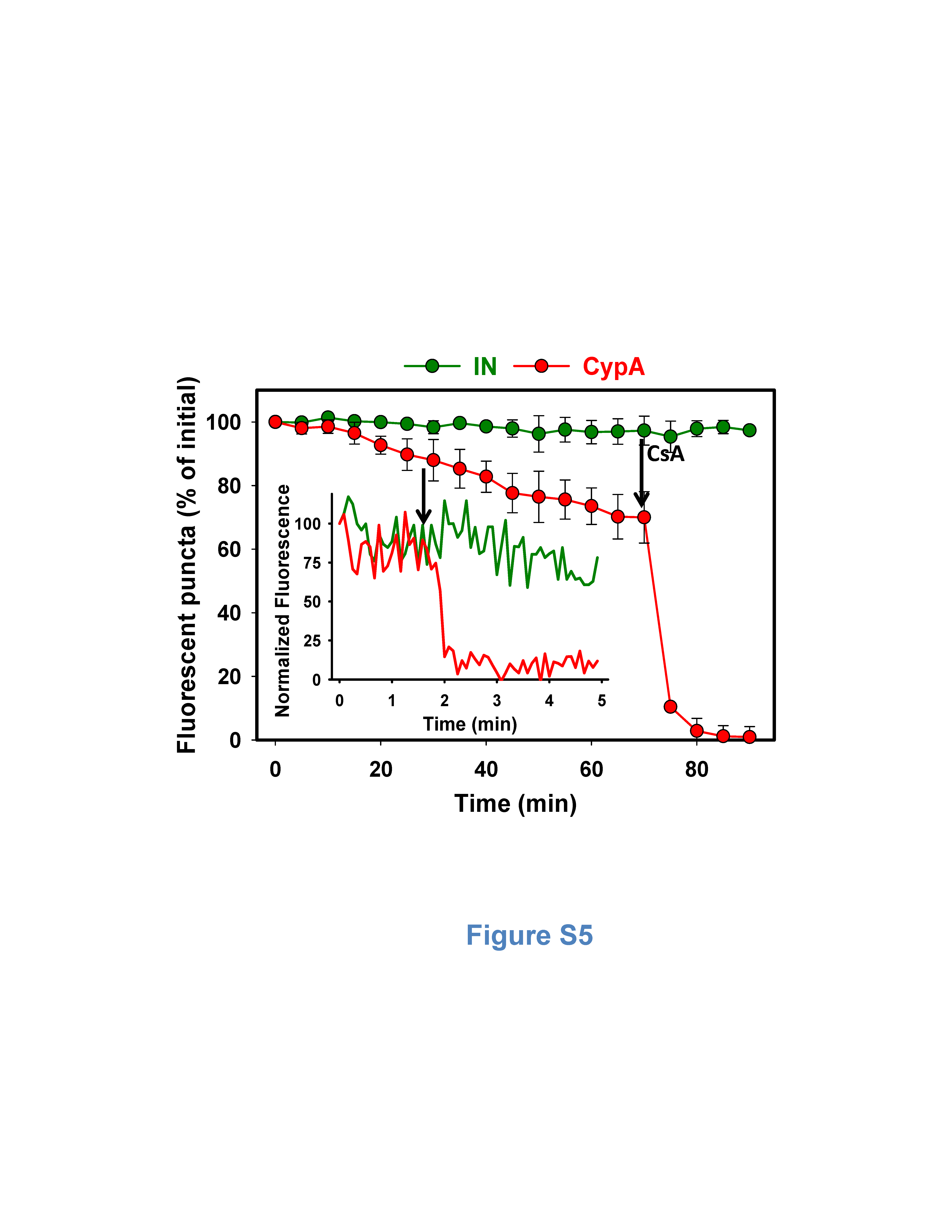

Supplement: S5 Fig — Same is in Fig 1E, but permeabilized particles were imaged at room temperature without fixation and CsA (5 μM) was added at 75 min. The number of CypA-DsRed puncta is plotted. Error bars are means and SD from 3 image fields. Inset: CypA-DsRed release from single permeabilized viral cores upon CsA addition (arrow). Mean fluorescence of IN-sfGFP-colocalized CypA-DsRed obtained by single particle tracking. (TIFF) [file ppat.1005709.s005.tiff]

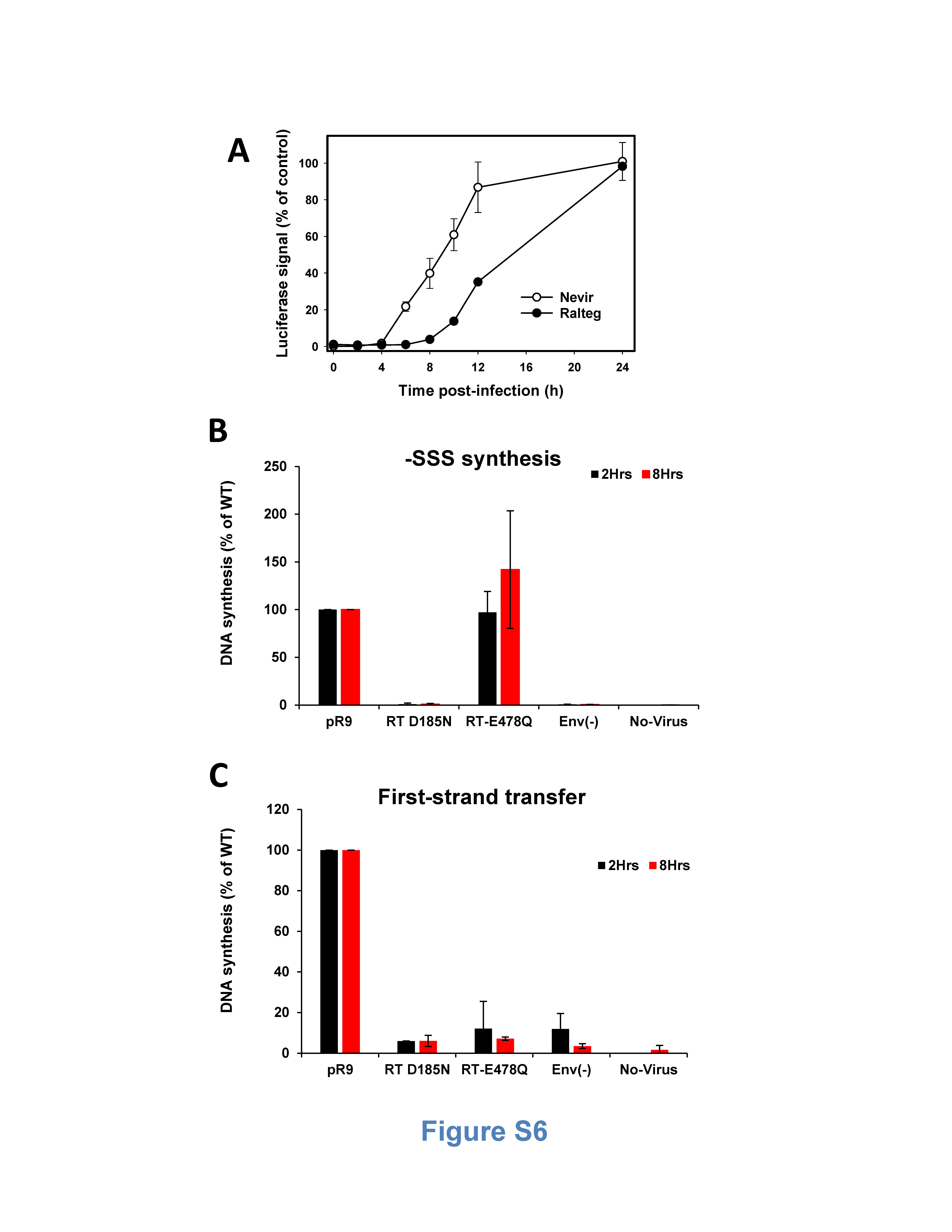

Supplement: S6 Fig — (A) Approximately 10,000 TZM-bl cells were infected in a 96 well plate with R9ΔEnv/VSV-G pseudotyped viruses, as described in Materials and Methods. Drugs targeting reverse transcription (Nevirapine, 10 μM) or integration (Raltegravir, 5 μM) were added to wells at indicated time points and maintained throughout the experiment (control cells were infected in the presence of respective amounts of DMSO). The resulting luciferase activity was measured at 48 h.p.i. The average luciferase activity from triplicate samples was measured. Error bars are SD. (B) and (C) Analysis of reverse transcription of RT mutants in target cells. Hela cells (100,000) were inoculated with DNase I-treated VSV-G-pseudotyped R9ΔEnv viruses (100 ng p24). After 2 hr and 8 hr, cells were detached with trypsin, pelleted, and extracts prepared for PCR as previously described (von Schwedler et al., J. Virol. 1993, 67:4945–4955). qPCR quantification of minus strand strong stop (panel B) and first strand-transfer reverse transcripts (panel C) were performed using stage-specific PCR primers and SYBR green detection. Strong Stop: Forward 5’-GGTCTCTCTGGTTAGACCA-3’; Reverse 5’-AAGCAGTGGGTTCCCTAGTTAG-3’. First Strand Transfer: Forward 5’-AGCAGCTGCTTTTTGCCTGTACT-3’; Reverse 5’-ACACAACAGACGGGCACACAC-3’. Standards consisting of plasmid DNA dilutions were used to generate a standard curve, and copy numbers of test samples determined by interpolation. Controls included cultures of cells inoculated with Env-defective HIV-1 particles lacking VSV-G (Env-) and cultures that were not exposed to virions (No virus). Mean and STD from 2 independent experiments performed in triplicates are shown. (TIFF) [file ppat.1005709.s006.tiff]

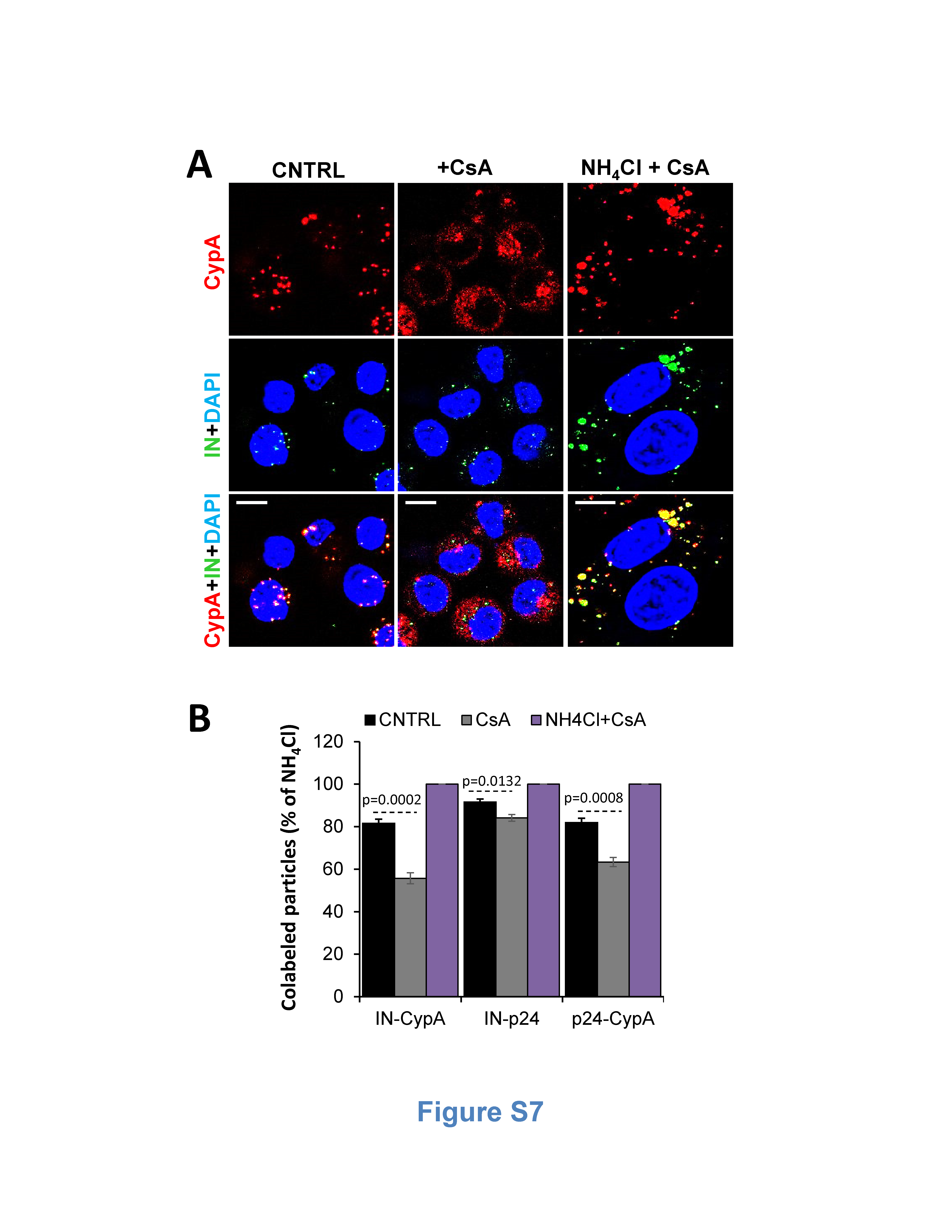

Supplement: S7 Fig — TZM-bl cells were infected with pR9ΔEnv-based pseudovirus co-labeled with IN-sfGFP and CypA-DsRed. Cells were incubated at 37°C for up to 2 h, at which time CsA (10 μM) was added and incubation continued for an additional 30 min before fixation and immunostaining for CA/p24. (A) Confocal images of the maximum intensity projection of the middle 3 Z-stacks are shown for control (untreated), 10 μM CsA-treated and CsA + 50 mM NH4Cl-treated cells. Top panel: CypA-DsRed alone; middle panel: IN-sfGFP+nuclear stain (2μg/ml Hoechst-33322); lower panel: Merged images of IN/CypA/nuclear stain. Scale bar 5 μm. (B) The fraction of IN-CypA, p24-CypA or IN-p24 co-labeled particles in CsA- and DMSO-treated samples is plotted after normalizing to IN-CypA colocalization in the NH4Cl+CsA treated control. Error bars represent standard error from 4 imaging fields of view. (TIFF) [file ppat.1005709.s007.tiff]

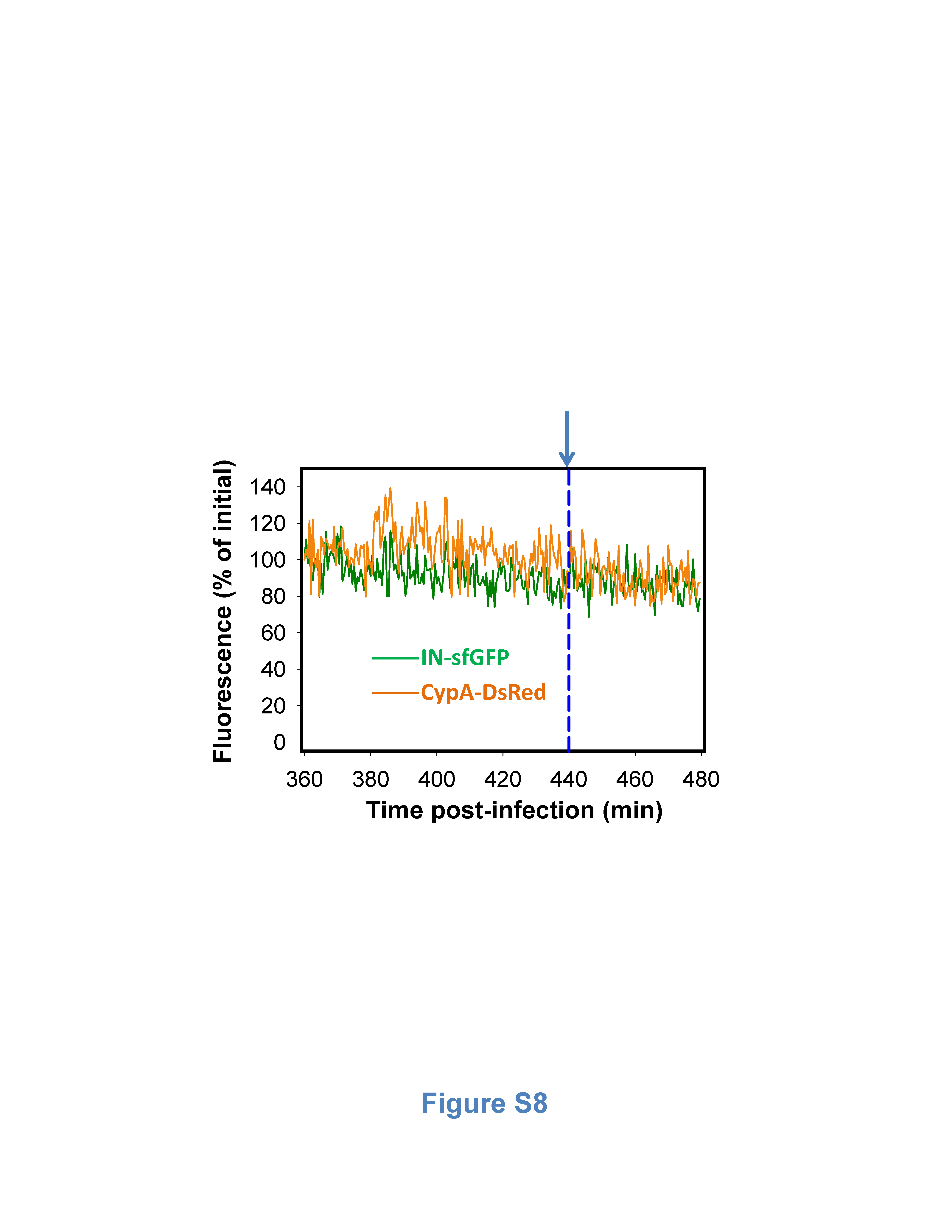

Supplement: S8 Fig — Image acquisition started at 6 h.p.i. and CsA was added 80 min after that (arrow). For details, see Fig 5C–5E. (TIFF) [file ppat.1005709.s008.tiff]

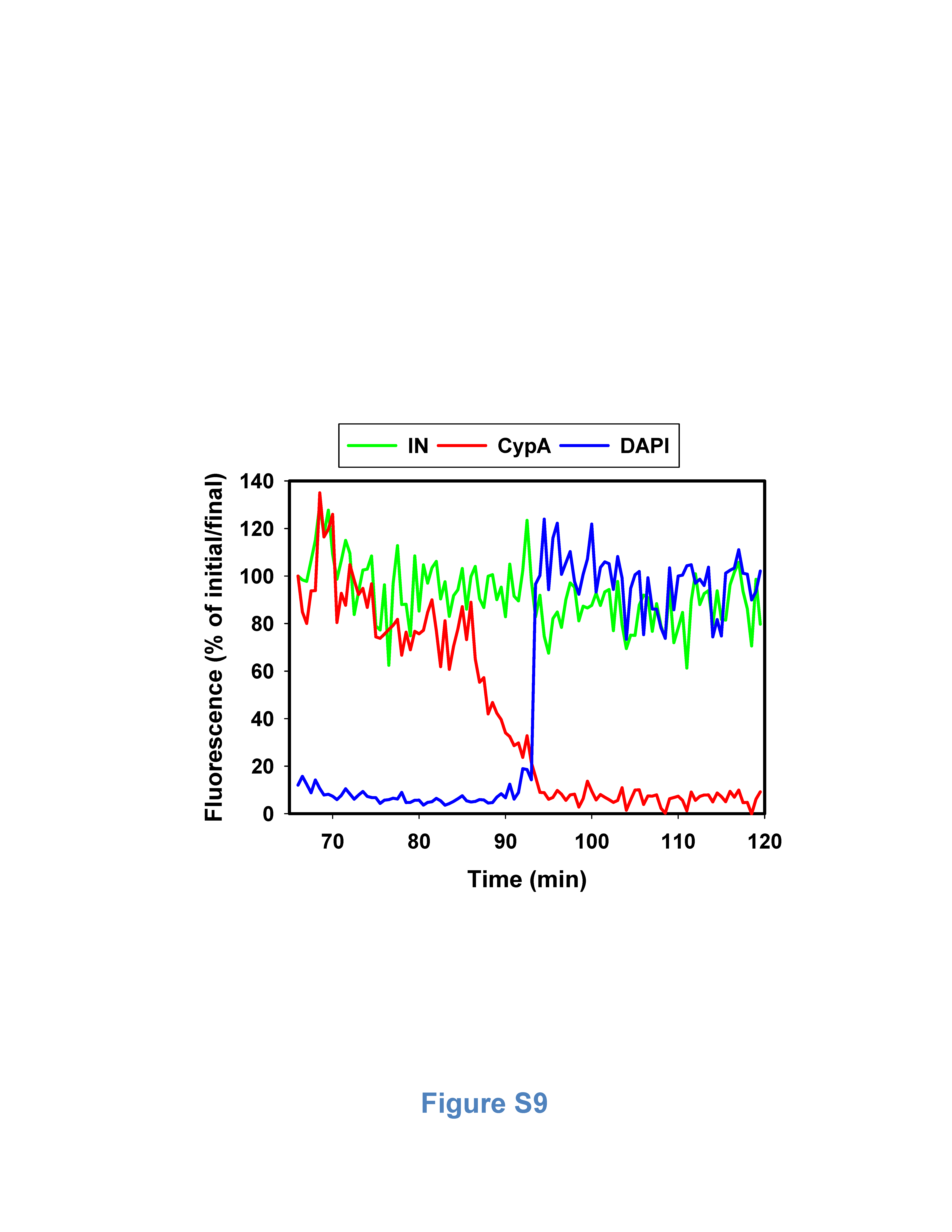

Supplement: S9 Fig — TZM-bl cells were inoculated with IN-sfGFP/CypA-DsRed labeled particles pseudotyped with VSV-G. Loss of the CA marker started at ~85 min post-infection and reached completion after the core approached the nucleus, as evidenced by the marked increase in the DAPI signal (blue) at ~93 min. Fluorescence signals are normalized to the initial values at 65 min (IN and CypA) or to the final value (DAPI). (TIFF) [file ppat.1005709.s009.tiff]
